# The proteome of developing barley anthers during meiotic prophase I

**DOI:** 10.1093/jxb/erab494

**Published:** 2021-11-10

**Authors:** Dominika Lewandowska, Jamie Orr, Miriam Schreiber, Isabelle Colas, Luke Ramsay, Runxuan Zhang, Robbie Waugh

**Affiliations:** 1 Cell and Molecular Sciences, The James Hutton Institute, Invergowrie, Dundee DD2 5DA, UK; 2 Information and Computational Sciences, The James Hutton Institute, Invergowrie, Dundee DD2 5DA, UK; 3 Division of Plant Sciences, University of Dundee, The James Hutton Institute, Invergowrie, Dundee DD2 5DA, UK; 4 School of Agriculture, Food and Wine, Waite Research Institute, University of Adelaide, Waite Research Precinct, Glen Osmond, SA 5064, Australia; 5 Ohio State University, USA

**Keywords:** Anthers, barley, *Hordeum vulgare*, meiotic prophase I, proteome, proteomics

## Abstract

Flowering plants reproduce sexually by combining a haploid male and female gametophyte during fertilization. Male gametophytes are localized in the anthers, each containing reproductive (meiocyte) and non-reproductive tissue necessary for anther development and maturation. Meiosis, where chromosomes pair and exchange their genetic material during a process called recombination, is one of the most important and sensitive stages in breeding, ensuring genetic diversity. Most anther development studies have focused on transcript variation, but very few have been correlated with protein abundance. Taking advantage of a recently published barley anther transcriptomic (BAnTr) dataset and a newly developed sensitive mass spectrometry-based approach to analyse the barley anther proteome, we conducted high-resolution mass spectrometry analysis of barley anthers, collected at six time points and representing their development from pre-meiosis to metaphase. Each time point was carefully staged using immunocytology, providing a robust and accurate staging mirroring our previous BAnTr dataset. We identified >6100 non-redundant proteins including 82 known and putative meiotic proteins. Although the protein abundance was relatively stable throughout prophase I, we were able to quantify the dynamic variation of 336 proteins. We present the first quantitative comparative proteomics study of barley anther development during meiotic prophase I when the important process of homologous recombination is taking place.

## Teaser

We performed a comprehensive proteomics study of accurately staged developing early meiotic anther in barley, providing new insight into the development of the male reproductive organ in small grain cereals.

## Introduction

In flowering plants, the development of male gametophytes occurs within the anthers of the stamens. Anthers contain a mixture of reproductive and non-reproductive tissues, and their development has been partitioned into three main phases: organ patterning and cell differentiation, meiosis, and post-meiotic pollen biogenesis (Goldberg *et al.*, 1993; Kelliher *et al.*, 2014). In the first phase, anther morphology is established through a process of cell and tissue differentiation, where a small proportion of reproductive generative cells or meiocytes are specified. During the meiotic phase, within the meiocytes, the parental chromosomes recombine to produce new combinations of alleles, followed by two rounds of division to generate haploid microspores. Post-meiosis, the haploid microspores differentiate and mature into pollen grains, a process accompanied by degeneration of the surrounding non-reproductive tissues that will ultimately lead to pollen release. The development of fertile anthers is therefore the result of a phased, spatially partitioned, and multistep process involving complex molecular and developmental interactions and temporal coordination.

The meiotic phase is particularly important in the context of plant reproduction and crop improvement through breeding. During meiosis, diversity is shuffled through the exchange of genetic materials by the process known as recombination. Recombination occurs during the first of the two meiotic divisions, and the new combinations of parental alleles occasionally release the new or improved traits that are subsequently the subject of breeder selection. In barley (*Hordeum vulgare*), male gametophytes (or meiocytes) develop within four pollen sacs inside the developing anthers. They have been estimated to comprise ~5–8% of anther tissues (Lewandowska *et al.*, 2019). The pollen sacs are surrounded by four layers of sporophytic anther tissues: tapetum, middle layer, endothecium, and epidermis (Gomez et al., 2015). The tapetum and the endothecium, in particular, are responsible for the nutrition and development of the pollen (Wang *et al.*, 2003; Ma, 2005; Parish and Li, 2010). Importantly, the successful progression of male meiosis depends not only on proteins expressed within the meiocytes, but also on those generated in the surrounding somatic tissues. Specification of the tapetal cell layer is critical for meiocyte maturation (Zhao *et al.*, 2002; Nonomura *et al.*, 2003; Yi *et al.*, 2012).

Most genome-wide studies aiming to address fundamental questions in anther development have focused on investigating variation in both transcript abundance and repertoire. The transcriptional landscapes have been studied through pollen development in many species including Arabidopsis (Yang *et al.*, 2011), maize (Yuan *et al.*, 2018), wheat (Feng *et al.*, 2017), sunflower (Flórez-Zapata *et al.*, 2014), and barley (Barakate *et al.*, 2021). However, given the general lack of correlation between transcript and protein abundance, our understanding of meiotic-phase tissue development should not be based solely on transcript abundance data. Furthermore, despite being generally assumed, there is only limited experimental evidence of germline protein-encoding transcripts being translated to functional proteins. As a result, the changing proteomic landscape, rather than the transcriptome, may in many cases be more informative of the important biology. Functional protein abundance is also the consequence of multilevel control. For example, translational control of existing mRNAs may have a key role, as shown in yeast where the elongation rate can dynamically regulate selective translation of stage-specific meiotic transcripts (Sabi and Tuller, 2019). Furthermore, recent studies indicate that anther-specific small RNAs, including miRNAs and phased small-interfering RNAs (phasiRNAs), regulate gene expression during male meiosis (Dukovic-Schulze et al., 2016; Bélanger *et al.*, 2020), and specific classes of phasiRNAs are enriched at different stages of meiosis in maize (Zhai *et al.*, 2015), rice (Komiya *et al.*, 2014; Fei *et al.*, 2016), wheat, and barley (Bélanger *et al.*, 2020).

Proteomics has the potential to improve our understanding of plant reproductive tissue development beyond the transcriptional level. However, studies on plant meiotic proteomes are limited, mainly due to the difficulty in obtaining sufficient reproductive tissue for detailed analysis. Outside of tissue-wide analyses of the proteomes of pooled anthers at various single developmental stages (Kim *et al.*, 2015; Ye et al., 2015, 2016; Li *et al.*, 2018), there are few reports investigating spatiotemporal changes in reproductive tissues at the protein level. One of the first studies explored proteome variation in rice anthers during male gametophyte development and detected 150 proteins that were differentially expressed in six discrete rice pollen developmental stages (Kerim *et al.*, 2003). Ischebeck *et al.* (2014) used a label-free quantitative approach to compare the proteomes of eight stages of tobacco pollen development and showed that the greatest changes in the protein machinery occur during the polarized microspore stage. Collado-Romero *et al.* (2014) investigated the proteome of isolated bulked rice meiocytes during prophase I progression, identified 1316 proteins in total, and classified them according to the developmental stage and putative functions). More recent research exploited a tandem mass tag (TMT) label-based quantitative proteomics approach to examine anther development in cotton at three different stages—meiosis, tetrad, and pollen maturation—of a male-sterile line (GA18) and its near-isogenic maintainer line (GA18M) with the objective of detecting male sterility-related proteins (Chen *et al.*, 2021). Yuan *et al.* (2018) investigated both the transcriptome and proteome of maize pre-meiotic anthers. They identified ~2800 proteins, including 56 meiotic proteins, with the combined data indicating that many of the latter were translated pre-meiosis (Yuan *et al.*, 2018).

In the current work, we aimed to discover how the anther proteome changes, both quantitatively and qualitatively, during meiotic prophase I when the important process of homologous recombination is taking place. One motivation was to test the sensitivity of our recently developed micro-proteomics pipeline for exploring spatiotemporal changes in the barley anther proteome (Lewandowska *et al.*, 2019). If successful, we anticipated that the approach would offer considerable added value over previous studies in that we are able to accurately cytologically stage anthers undergoing meiotic prophase I. This would potentially find application in subsequent comparative studies between wild-type and mutant plants where meiosis or anther development was somehow disturbed (e.g. Colas *et al.*, 2017). Here we analysed five replicates of six consecutive and accurately staged developmental time points of anthers containing meiocytes spanning the whole of prophase I, a developmental process taking 36–48h. All three 0.6–1.1mm anthers were dissected from individual spikelets from the middle of developing barley inflorescences. Meiosis was staged immunocytologically using one anther, and the protein composition of the remaining two was identified and quantified using label-free high-resolution mass spectrometry (MS). We identified >6100 proteins, including 82 known and putative meiotic proteins, and were able to quantify dynamic variation in the proteome during barley anther development. We believe this comprehensive proteomics study has generated one of the largest datasets related to accurately staged developing early meiotic anther in plants and provides a new perspective on our understanding of the development of the male reproductive organ in small grain cereals.

## Materials and methods

### Materials

Complete protease inhibitor cocktail tablets were from Roche. NuPAGE gels, running buffer, LDS buffer, and sample reducing agent were from Thermo Scientific. InstantBlue staining kits were from Expedeon. Trypsin was from Promega. Anti-TaASY1 (rabbit) antibodies were from Agrisera and anti-rabbit Alexa Fluor® (568) antibodies from Invitrogen. Fluoroshield mounting medium for slides was from Abcam. The Pepmap 100 C18 columns were from Thermo Scientific. All other materials were obtained from Sigma.

### Plant material and sample collection

We used barley (*H. vulgare*) cv. Golden Promise throughout. The seeds were obtained from The James Hutton Institute seed store. Plants were grown in 70% humidity under 16h of light at 18–20 °C and 8h of dark at 16 °C until they reached meiosis (6–7 weeks) in a controlled-environment growth room. For anther collection, 0.8–1.4cm spikes (i.e. developing barley inflorescences) were collected and anthers within a size range of 0.6–1.1mm were dissected manually from individual developing spikelets with insulin syringes on a plastic Petri dish under a stereoscopic microscope (with graticule). As meiosis is not perfectly synchronized along the length of the spike, we only collected anthers from the middle spikelets. Each barley spikelet contains three developmentally synchronized anthers and so meiosis was monitored in one anther from each spikelet by immunostaining using meiosis-specific antibodies. The remaining two anthers were placed on a cavity glass slide in a drop of 1× phosphate-buffered saline (PBS) including protease inhibitor cocktail prepared according to the manufacturer’s instructions, and squashed with an insulin needle. Samples were placed immediately in an Eppendorf tube containing LDS NuPage buffer including 1/10th volume of NuPAGE sample reducing agent. Six sizes of anthers were collected: 0.6, 0.7, 0.8. 0.9, 1.0, and 1.1mm, each in five biological replicates (where each biological replicate was an individual plant). In total, we used 10 anthers for the proteomic analysis of each meiotic stage.

### Sample preparation by 1D SDS–PAGE gel fractionation

Individual paired squashed anthers were treated according to Lewandowska *et al.* (2019). Briefly, paired anthers from the same spikelet were put into an Eppendorf tube and extracted with the LDS NuPage buffer. Size fractionation of protein extracts was then achieved by quick SDS–PAGE analysis on 4–12% (w/v) Bis-Tris NuPage gels (Thermo Scientific) using MES running buffer (50mM MES, 50mM Tris base, 0.1% SDS, 1mM EDTA, pH 7.3). Electrophoresis was run for only 10–15min at 200 V constant. Gels were stained with InstantBlue according to the manufacturer’s instructions (Expedeon). Each gel track was cut into three fractions corresponding to 3–17, 17–62, and 62–250kDa. Gel pieces were de-stained by immersion in 50% acetonitrile, and proteins were reduced by incubation with 10mM DTT in 20mM ammonium bicarbonate and alkylated with 50mM iodoacetamide in 20mM ammonium bicarbonate by incubating for 30min in the dark. The gel slices were then digested with trypsin 12.5 μg ml^–1^ in 20mM ammonium bicarbonate overnight at 30 °C, shaking at 300rpm on an Eppendorf Thermomixer shaker, and peptides were extracted the next day by incubating the gel pieces in 50% acetonitrile. Samples were dried to ~10 μl by vacuum centrifugation in an Eppendorf speedvac at room temperature.

### Immunocytology and microscopy

One anther from each spikelet was fixed in 4% formaldehyde (1× PBS/0.5% Triton X-100/0.5% Tween-20) for 20min, rinsed once in 1× PBS, and transferred onto a Polysine® 2 (poly-l-lysine coated) slide. Then the anther was squashed gently with an insulin needle and left to air dry. Immunocytology was done according to Colas *et al.* (2017). Briefly, the primary antibody solution consisted of anti-TaASY1 (rabbit) at 1:1000 in 1× PBS blocking buffer. Primary antibodies were added on the slide, incubated in a wet chamber for 30min at room temperature, followed by up to 36h at 4–6 °C. Slides were warmed for 1h at room temperature before washing for for 15min in 1× PBS and incubating for 90min at room temperature in the secondary antibody solution consisting of anti-rabbit Alexa Fluor® (568) antibodies (Invitrogen) diluted in 1× PBS blocking buffer (1:600). Slides were washed for 15min in 1× PBS and mounted in Fluoroshield Mounting Medium containing DAPI (Abcam). 3D confocal stack images were acquired with a LSM-Zeiss 710 using laser light (405nm and 561nm) sequentially with minimum pinhole opening, two lines averaging. Raw images were processed with Imaris deconvolution ClearView 9.5. Projections of 3D pictures, and light brightness/contrast adjustment were performed with Imaris 9.5.1 (Bitplane).

### LC-MS/MS and MaxQuant analysis, and label-free protein quantification

An Ultimate 3000 RSLC nano system (Thermo Scientific) was used with 15 µl of peptides (in 1% formic acid) injected onto an Acclaim PepMap 100 (C18, 100 µm×2cm) nano-trap column (Thermo Scientific). After washing with 2% (v/v) acetonitrile in 0.1% (v/v) formic acid, peptides were resolved on a 50 cm×75 µm Easy-Spray PepMap RSLC C18 column (Thermo Scientific) over a 200min organic gradient with a flow rate of 300 nl min^–1^. The chromatography performed for these samples was as follows. The gradient commenced with 6min of 95% buffer A (0.1% formic acid)/5% buffer B (80% acetonitrile, 0.08% formic acid), followed by a linear gradient to 35% buffer B over 130min, then an increase to 98% buffer B for 22min duration, and completed with a return to 2% buffer B at 153min for 17min. Ions accepted for MS/MS were 2+ and greater. Dynamic exclusion was set to 45s, and the inclusion mass width for precursor ions was 10ppm. Peptides were transferred to the mass spectrometer via an Easy-Spray source with temperature set at 50 °C and a source voltage of 2.0kV. Tandem MS analysis was carried out on a Q Exactive Plus Mass Spectrometer (Thermo Scientific) using data-dependent acquisition, measuring and sequencing the top 15 ions.

The resulting raw files were processed and searched using MaxQuant version 1.6.6.0 and the Andromeda peptide search engine (Cox *et al.*, 2011; Cox and Mann, 2008), against a custom-made *H. vulgare* Barley Anther Proteome Database (BaAP-Db), containing 25 776 protein sequences (see below). Enzyme specificity was set as trypsin and the number of missed cleavages permitted was 2. The variable modifications were set as oxidation of methionine and acetylation of the protein N-terminus. Fixed modifications were set to carbamidomethylation of cysteines only. The MS tolerance was set to 7ppm, with the MS/MS tolerance set to 0.5Da. The ‘match between runs’ function was enabled between corresponding fractions. The peptide and protein false discovery rates (FDRs) were both set to 1% (Cox and Mann, 2008), and the proteins used for further analysis had two or more peptides assigned to them. Relative protein quantification was performed with MaxQuant’s LFQ algorithm, which combines and adjusts peptide intensities into a protein intensity value; the ‘stabilize large LFQ ratios’ function was enabled.

For the methylation analysis, a new MaxQuant search was done with the same parameters as above but with methylation of arginine and lysine defined as variable modifications.

The MS proteomics data have been deposited to the ProteomeXchange Consortium via the PRIDE (Perez-Riverol *et al.*, 2019) partner repository with the dataset identifier PXD026200.

### Barley Anther Proteome Database (BaAP-Dd)

We generated a genotype-specific ‘Golden Promise’ anther/meiocyte proteome dataset using RNA-seq data previously published by Barakate *et al.* (2021) (PRJNA558196). First, a transcriptome combining long PacBio and short Illumina reads was built as described by Barakate *et al.* (2021). Second, a protein dataset was produced by using NCBI ORF finder on all transcripts, followed by a BLAST search against the NCBI non-redundant protein database using diamond blastp search [--query-cover 60 --evalue 1e-30]. The longest protein per gene with a blast hit was extracted to build the final protein database which contained 25 776 unique protein sequences.

### Data processing and differential protein abundance analysis

We used the R package Proteus (https://github.com/bartongroup/Proteus, version 0.2.14) (Gierlinski *et al.*, 2018, Preprint) for downstream protein and peptide differential expression analysis. A filtering step for protein abundance was added, only keeping samples with intensities in at least three of the five replicates in at least one sample. This left 3120 proteins for the final analysis. Each time point was compared in a pairwise manner with all other time points to identify differentially abundant proteins (DAPs).

### Protein function and Gene Ontology analysis

Annotation [protein description and Gene Ontology (GO) term identification] of the BaAP proteins was added by using PANNZER2 (http://ekhidna2.biocenter.helsinki.fi/sanspanz/, Törönen *et al.*, 2018). GO enrichment analysis was done with the R package topGO using the set of proteins generated from our previously published anther/meiocyte transcriptomics (Barakate *et al.*, 2021) as a background and the fisher weight01 algorithm, and a *P*-value cut-off below 0.001 (Alexa *et al.*, 2006).

### Figures for AGO protein abundance and AGO gene expression

To compare the pattern of Argonaute (AGO) transcript abundance in the Barley Anther Transcriptome (BAnTr) (Barakate *et al.*, 2021) with BaAP AGO protein abundance, the datasets were combined using tidyr (v1.0.2; Wickham and Henry, 2020). Heat maps were prepared using ggplot2 (v3.3.0; Wickham, 2016), cowplot (v1.0.0; https://wilkelab.org/cowplot/), and viridis (v0.5.1; https://github.com/sjmgarnier/viridis). For box plots, BaAP anther proteins in all samples were binned according to their lengths indicating meiotic stage relative to the BAnTr dataset (Barakate *et al.*, 2021) as follows: 0.6 mm=pre-meiotic, 0.7–0.9 mm=leptotene–zygotene, 1.0 mm=pachytene–diplotene, and 1.1 mm=metaphase I. Box plots were generated using ggplot2 (v3.3.0; Wickham, 2016), cowplot (v1.0.0; Wilke, 2019), and viridis (v0.5.1; Garnier, 2018). The corresponding R code is available on GitHub www.github.com/BioJNO/BaMP).

## Results

### A developmental time series of meiotic-stage barley anthers

In barley cv. Golden Promise, meiosis occurs 6–7 weeks after sowing seeds and progresses over a period of ~36–48h. To quantify changes in protein abundance during meiotic progression, we dissected anthers from individual barley spikelets at six different meiotic stages, represented by anther lengths of 0.6, 0.7, 0.8, 0.9, 1.0, and 1.1mm. As we had previously shown a strong correlation between meiotic stage and anther length (Arrieta *et al.*, 2020), we assumed that anthers of 0.6–1.1mm would be in meiotic prophase I. However, as growing conditions can sometimes influence this correlation, we performed an additional control using immunocytology. Anthers were dissected from the middle of the developing inflorescence under a stereomicroscope, measured, and collected for protein extraction according to size. As each spikelet contains three developmentally synchronized anthers, we used one for meiotic staging by immunocytological staining with antibodies against the HORMA domain protein ASY1 (Colas *et al.*, 2017) and the remaining two were set aside for proteomics analysis. In total, five biological replicates of paired anthers were collected for each time point/size.

**Fig. 1.**
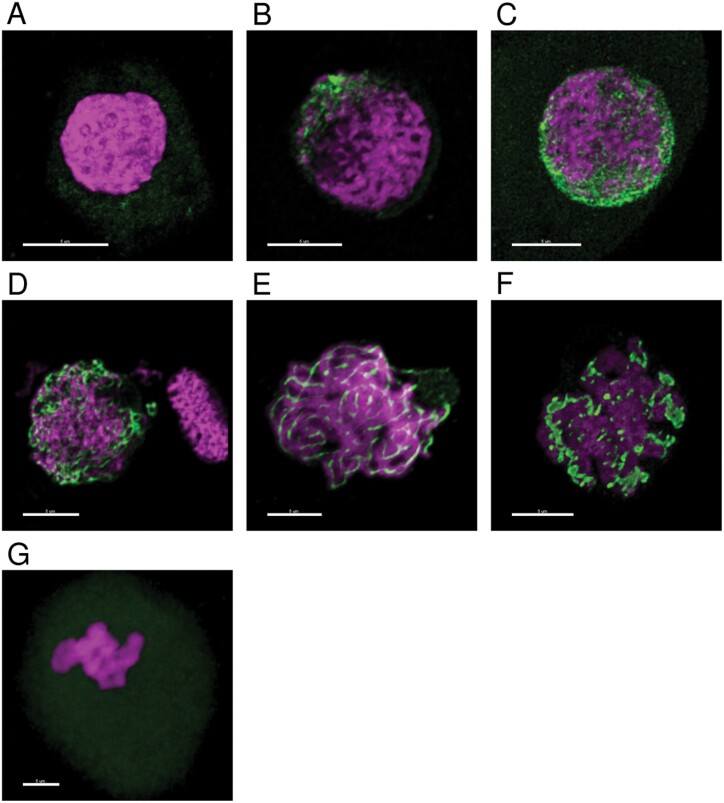
Anther staging using immunolocalization with DAPI (magenta) and anti-ASY1 antibody (green). (A) 0.6mm, pre-meiotic stage; (B) 0.7mm, early leptotene; (C) 0.8mm, leptotene/early zygotene; (D) 0.9mm, zygotene; (E) 1.0mm, pachytene; (F) 1.0mm, diplotene; (G) 1.1mm, metaphase I. Scale bar 5 µm.

Figure 1 shows representative immunostaining of meiocytes present in each anther size, confirming the correlation between size and meiotic stage. Spreads of 0.6mm anthers showed very weak labelling with anti-ASY1 in the cytoplasm, typical of the pre-meiotic stage (Fig. 1A). The consecutive time point of anther size 0.7mm was staged as early leptotene, identified by a spotty ASY1 signal that starts to cluster in the periphery of the nucleus (Fig. 1B), corresponding to the telomere cluster (Higgins *et al.*, 2012). At later stages of leptotene, ASY1 labelling covers the whole nucleus and linear ASY1 axes are formed at the initiation of synapsis (anther size 0.8mm) (Fig. 1C) as previously described (Colas *et al.*, 2017; Barakate *et al.*, 2021). By 0.9mm, anthers had fully entered the zygotene stage characterized by thicker chromatin threads stained by DAPI and a slightly weaker ASY1 signal reflecting synapsis progression (Fig. 1D). At 1.0mm, we observed a range of pachytene–diplotene stages (Barakate *et al.*, 2021). At pachytene (Fig. 1E), synapsis was complete, with discontinuous ASY1 signals and more open chromatin threads, while diplotene was characterized by the presence of a tinsel-like structure as a result of extensive chromosome axis remodelling (Fig. 1F) (Colas *et al.*, 2017). Prophase I was concluded when anthers reached 1.1mm in length and we could identify the presence of seven ring bivalents (Fig. 1G), characteristic for metaphase I. Thus, our developmental time series comprised anthers containing meiocytes at six different meiotic stages covering the entirety of prophase I. We prepared and analysed protein extracts from all 30 samples.

### Barley Anther Proteome (BaAP)

We deployed our previously described ‘micro-proteomic’ workflow (Lewandowska *et al.*, 2019) to characterize the proteome of all 30 biological samples. To improve identification of phase-dependent anther-specific proteins we searched our proteomics data against a custom-made protein database generated from a reference Barley Anther Transcriptome (BAnTr) (Barakate *et al.*, 2021) based on Golden Promise, the same genotype as was used here. This newly developed BaAP-Db (Barley Anther Proteome Database) contained 25 776 protein sequences and offered improved search sensitivity, specificity, and significantly higher sequence coverage, when compared with the Uniprot *H. vulgare* database (from March 2017) that we have previously used. The MS data covered, on average, 30.24% of each protein sequence when the BaAP-Db was used compared with 24.73% when the analysis was run against the Uniprot database.

**Fig. 2.**
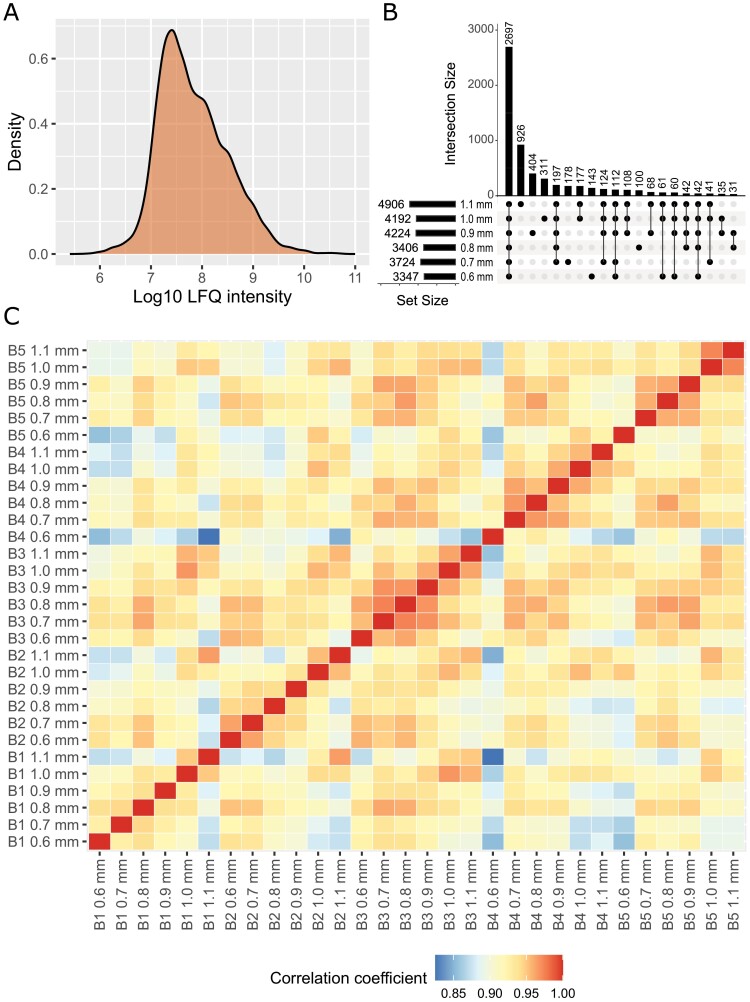
Summary of the Barley Anther Proteome (BaAP). (A) Distribution of the protein intensities in 30 samples. (B) Intersect plot of proteins identified in anthers representing different developmental time points. The combination matrix at the bottom of the plot indicates comparison-specific and shared subsets, and the bar above it shows their sizes. The set size of each comparisons is shown on the left. (C) Pearson correlation coefficients of LFQ intensities of all identified proteins across six developmental time points and five biological replicates.

Across the six developmental time points, a total of 73 804 peptides were identified, representing 6431 proteins (6105 with two or more peptides), with between 3300 and 4900 proteins in each time point. By merging all 6431 non-redundant proteins from all 30 samples, we built a comprehensive Barley Anther Proteome (BaAP). The dynamic range of protein expression in BaAP covered six orders of magnitude (Fig. 2A). Most proteins were identified with 2–10 peptides (56%), while 39% of the BaAP was identified with >10 peptides (Supplementary Fig. S1). The most abundant proteins, identified with >100 peptides, included acetyl-CoA carboxylases involved in fatty acid biosynthesis, pre-mRNA splicing proteins, translational activator, E3 ubiquitin-protein ligase, nuclear pore protein, and clathrins, indicating key processes in the developing anther as lipid biosynthesis, gene expression regulation, control of protein homeostasis and localization, and intracellular trafficking.

Within the identified proteins, 2697 were detected in all six developmentally staged samples, while 3734 were shared between some or detected only at specific individual time points (Fig. 2B). All samples were analysed as five biological replicates to obtain high-quality data. The Pearson correlation coefficients (*R*^2^) for all replicates in the same anther size/stage was consistently higher than 0.88, confirming the high reproducibility of the approach (Fig. 2C).

To obtain a better insight into the roles of the 6431 identified BaAP proteins, we first performed high-throughput functional annotation with PANNZER2 (Törönen *et al.*, 2018). Each of the identified proteins has been given a predicted function and assigned to one or more GO classes (Supplementary Table S1.). Then we carried out GO enrichment analysis of the BaAP in comparison with the set of proteins generated from our previously published anther/meiocyte transcriptomics data (Barakate *et al.*, 2021) using the topGO R package (Alexa and Rahnenfuhrer, 2020; Supplementary Table S2; Supplementary Fig. S2A). The most significant (*P*<1e-30) molecular function included ‘structural constituent of ribosome’ as the top category, followed by ‘mRNA, cobalt ion and GTP binding’, ‘GTPase activity’, ‘translation initiation factor activity’, and others. The top enriched biological processes (*P*<1e-30) consisted of ‘translation’, ‘intracellular and ER to Golgi vesicle-mediated transport’, ‘glycolytic process’, ‘response to cadmium ion’, and a range of categories connected to RNA processing (‘rRNA processing’, ‘regulation of alternative mRNA splicing’, and ‘mRNA transport’). GO enrichment indicates that protein biosynthesis, intracellular transport, and energy generation, mechanisms essential for somatic cell growth and development, comprise the major biological processes in the developing meiotic anther.

### Quantitative protein abundance in early developing meiotic-stage barley anthers

To identify DAPs, we quantified and analysed all 6431 proteins identified using the R package Proteus (Gierlinski *et al.*, 2018, Preprint). First, we removed replicate 5 from the 0.6mm sample for all further analysis as it was identified on the principal component analysis (PCA) test as an outlier (Supplementary Fig. S3, arrow). A filtering step was added to the analysis to remove proteins with too much missing data, only keeping those with intensities in at least three out of five replicates in any stage. After filtering, of the 6431 detected proteins, 3120 proteins remained. This group of 3120 proteins was used to identify DAPs. Proteins were considered differentially abundant (DAPs) when the log_2_ fold change (LFC) in abundance between any stages was ≥1 or below –1 and the corrected *P*-value was <0.05 in a pairwise comparison of any two stages. We set up comparisons among all possible combinations of stages; 15 comparisons in total. Using these criteria, 336 proteins were determined to be DAPs (Supplementary Table S3). Thus, over all sampled stages, ~5% of the identified proteins changed in abundance by ≥2-fold between any two stages.

Then we implemented GO enrichment analysis of the 336 significantly DAPs across all stages, as described above. As expected, GO enrichment analysis points to an important role for DNA replication and repair during prophase I, with the top enriched biological processes centred around GO terms related to ‘pre-replicative complex assembly involved in cell cycle DNA replication’, ‘double-strand break repair via break-induced replication’, ‘DNA replication initiation’, ‘mitotic DNA replication’, ‘DNA strand elongation involved in DNA replication’, and ‘gene silencing by RNA’ (Supplementary Fig. S2B; Supplementary Table S3). Other top enriched biological processes included ‘glycolytic process’, ‘phosphorus metabolic process’, and ‘peptidyl-asparagine modification’, reflecting some of the fundamental mechanisms providing energy and nutrients required for somatic cell growth in the dynamically developing anther.

We were surprised to find ‘response to cadmium ion’ as another enriched GO term within DAPs. However, anther development GO terms are often associated with those connected to cadmium/copper response, and this can be explained by the fact that cadmium exposure induces DNA damage in multiple plant species and, consequently, alters gene expression of DNA repair genes (Huybrechts *et al.*, 2019).

To further investigate differential protein abundance across developing meiotic anthers, we checked all stage comparisons in terms of the number of identified DAPs. Not surprisingly, the highest number of DAPs was observed when comparing the proteome of anthers in pre-meiotic stage (size 0.6mm) with anthers that reached the end of prophase I (size 1.1mm). Notably, 170 DAPs (Supplementary Table S4) detected for this developmental transition equals half of all identified DAPs across all stages. The most up-regulated proteins belonged to a class of enzymes involved in glycolysis, fatty acid and flavanol biosynthesis, and photosynthesis, highlighting the requirements of the actively growing anther for ‘high energy’ molecules, lipids, and carbon compounds. However, overall, our data suggest that relative protein abundance in anthers is rather stable throughout prophase I as we did not detect large quantitative changes between consecutive stages. The most significant switch when looking at subsequent stages occurs at the comparison of 0.9mm versus 1.0mm anthers (transition from zygotene to pachytene–diplotene), with 58 DAPs identified (Supplementary Table S5).

### Transition from zygotene to pachytene–diplotene

Anther growth from 0.9mm to 1.0mm marks an important cell cycle advancement in the developing meiocytes from zygotene to pachytene and diplotene, where the synaptonemal complex disassociates to prepare the chromosome for segregation. We therefore explored whether the observed protein abundance changes were likely to reflect changes in the small proportion of meiotic cells in the developing anther as they progress from the pre-crossing over to post-crossing over stages.

We identified 58 DAPs in this transition (Supplementary Table S5). The most up-regulated DAP was an orthologue of the known meiotic AGO protein MEL1 (Meiosis Arrested at Leptotene 1) (Nonomura *et al.*, 2007) that changed in abundance by 4-fold (LFC >2). Other highly up-regulated proteins detected in the anther during the switch from zygotene to pachytene/diplotene could be organized into several groups associated with the following biological processes: carbohydrate and lipid metabolism, oxidoreductase activity, and photosynthesis. There were 13 down-regulated proteins, including meiotic HORMA protein ASY1. Several down-regulated DAPs function in DNA unwinding and replication, including six DNA helicases. Three of these are members of the minichromosome maintenance protein complex (MCM), namely MCM7, MCM5, and MCM3, which are responsible for the separation of duplex DNA during replication (Ortega *et al.*, 2012; Sonneville *et al.*, 2012; Hatkevitch et al., 2021).

GO enrichment analysis supported down-regulated proteins in this transition as mainly belonging to the DNA helicase group, involved in various aspects of DNA replication and double-strand break repair (Knoll and Puchta, 2011). The only two up-regulated GO terms were: hydroxymethyl-, formyl-, and related transferase activity, and sporopollenin biosynthetic process (Supplementary Fig. S2C; Supplementary Table S5). Hydroxymethyl transferases are the main source of activated one-carbon units available to the cell and crucial for cell proliferation (Ruszkowski *et al.*, 2018). Sporopollenin is a biopolymer comprising the outer shell of plant pollen (Mackenzie *et al.*, 2015).

### Identification and quantification of meiosis-related proteins

Two of the primary aims of our study were to understand how the protein composition of developing anthers changes during different stages of early meiosis and whether the micro-proteomics approach we described previously and adopted here had sufficient sensitivity to assess changes in the meiocyte proteome. We recognized that this could be a challenge as we had previously estimated that developing meiocytes comprised only ~5–8% of the anther cells (Lewandowska *et al.*, 2019). Still, within the collection of 6431 detected BaAP proteins revealing a six order of magnitude variation in abundance, we identified 82 proteins as known or putative meiotic proteins (Table 1), representing every stage of early meiosis.

**Table 1.** Putative meiotic proteins identified in the Barley Anther Proteome

BaAP number	Meiotic protein	DAP	NP	*Q*-value
**BaAP.GP.2HG0912**	**HvAGO5b/MEL1**	+	49	0
BaAP.GP.4HG0241	HvAHP		6	0
BaAP.GP.2HG1933	HvAML2		4	0
BaAP.GP.7HG0009	HvAML3		7	0
**BaAP.GP.5HG2173**	**HvASY1**	+	26	0
BaAP.GP.2HG2224	HvATK_a		3	0.008694
BaAP.GP.2HG3329	HvATK_b		4	0
BaAP.GP.UnG0003	HvATM		13	0
BaAP.GP.1HG2380	HvBRCA1		2	0.000177
BaAP.GP.4HG2013	HvBUB3a		19	0
BaAP.GP.2HG1450	HvCHD4		15	0
BaAP.GP.1HG2929	HvDDB1		55	0
BaAP.GP.4HG0309	HvDDM1A		24	0
BaAP.GP.5HG0973	HvDMC1		4	0
BaAP.GP.5HG0795	HvFIGL		1	0.000996
BaAP.GP.7HG1475	HvFIP37		7	0
BaAP.GP.3HG1959	HvFVE		25	0
BaAP.GP.2HG1619	HvHEN1		25	0
BaAP.GP.6HG1643	HvHOP2		50	0
BaAP.GP.4HG1671	HvHSP70		41	0
BaAP.GP.7HG1521	HvHSP90.2		63	0
BaAP.GP.5HG2025	HvHSP90.3		73	0
BaAP.GP.5HG1970	HvHSP90.5		43	0
BaAP.GP.5HG0753	HvHSP90.6		48	0
BaAP.GP.7HG3617	HvHSP90.7	+	71	0
BaAP.GP.4HG1256	HvINO80		17	0
BaAP.GP.1HG0518	HvISW2_A		57	0
BaAP.GP.3HG0363	HvISW2_B		53	0
BaAP.GP.5HG0314	HvKU70		20	0
BaAP.GP.UnG1116	HvKU80	+	29	0
BaAP.GP.1HG1105	HvLIG1		40	0
**BaAP.GP.3HG2543**	**HvMAD1**	+	22	0
BaAP.GP.2HG2382	HvMAD2		4	0
BaAP.GP.5HG0630	HvMCM7	+	56	0
BaAP.GP.4HG0649	HvMEI1		3	0.000176
BaAP.GP.2HG1615	HvMET1_A		78	0
BaAP.GP.4HG2462	HvMETTL14		9	0
BaAP.GP.5HG1377	HvMND1		6	0
BaAP.GP.3HG1181	HvMOS4/BCA52		10	0
BaAP.GP.7HG0556	HvMPA		56	0
**BaAP.GP.2HG3622**	**HvMRE11A**		15	0
**BaAP.GP.1HG0781**	**HvMSH2**		12	0
BaAP.GP.2HG2453	HvMSH3		1	0.004612
**BaAP.GP.5HG1649**	**HvMSH6**		3	0
BaAP.GP.6HG1771	HvMTA		6	0
**BaAP.GP.1HG1060**	**HvNBS1**		7	0
BaAP.GP.3HG2271	HvNIH	+	50	0
BaAP.GP.2HG2334	HvPCH2		13	0
BaAP.GP.2HG2526	HvPCNA		21	0
BaAP.GP.4HG0704	HvPS1		4	0
BaAP.GP.7HG1273	HvRAD23A		11	0
BaAP.GP.5HG0552	HvRAD50		44	0
BaAP.GP.3HG2547	HvRAD52-1	+	14	0
BaAP.GP.7HG1724	HvRBR1		7	0
BaAP.GP.UnG0824	HvRCC2		29	0
BaAP.GP.2HG0466	HvRECQL3b		19	0
BaAP.GP.7HG2732	HvRFC1		47	0
BaAP.GP.UnG0621	HvRFC5	+	21	0
BaAP.GP.6HG2386	HvRPA1A		10	0
BaAP.GP.4HG2016	HvRPA1B		22	0
BaAP.GP.6HG2840	HvRPA2A	+	11	0
**BaAP.GP.6HG1523**	**HvRPA2B**		4	0
**BaAP.GP.7HG3028**	**HvRPA2C**		4	0
BaAP.GP.3HG1110	HvRPA3B		2	0
BaAP.GP.2HG1823	HvSAD2		36	0
BaAP.GP.2HG1742	HvSCC2		36	0
BaAP.GP.4HG1035	HvSCC3		32	0
BaAP.GP.6HG1341	HvSET		18	0
**BaAP.GP.7HG2957**	**HvSMC1**		16	0
BaAP.GP.3HG2853	HvSMC2		67	0
BaAP.GP.1HG2902	HvSMC3		73	0
**BaAP.GP.5HG1276**	**HvSMG7**		16	0
BaAP.GP.4HG1599	HvSPO11-3		12	0
BaAP.GP.3HG1572	HvSUMO1		18	0
BaAP.GP.3HG1209	HvSUN1/2		23	0
BaAP.GP.1HG0447	HvSUN1/2		14	0
BaAP.GP.6HG1890	HvTOPIIA		78	0
BaAP.GP.1HG0898	HvTranslin		14	0
BaAP.GP.3HG1871	HvXPB2		3	0
BaAP.GP.1HG0438	HvXPD		5	0
**BaAP.GP.5HG2826**	**HvXRCC4**		10	0
**BaAP.GP.2HG2203**	**HvZIP1**		10	0

DAP, differentially abundant protein (proteins identified as DAPs are marked with ‘+’); NP, number of matched peptides; *Q*-value, the ratio of reverse to forward protein groups generated by MaxQuant software. It operates as the *P*-value, where smaller is more significant and reflects better match quality. Proteins not detected in pre-meiotic anthers are in bold.

The two most abundant meiotic proteins were DNA topoisomerase 2 (TOP2A) (Martinez-Garcia *et al.*, 2018) and DNA (cytosine-5)-methyltransferase (MET1_A) (Yelina *et al.*, 2015), which were both identified with 78 peptides. Members of the HSP90 (heat shock protein 90) and SMC (structural maintenance of chromosome) families, components of ISWI chromatin-remodelling complex, and DNA replication licensing factor MCM7 were also highly abundant. MSH3 and FIGL1 were identified with a single peptide and therefore were not included in the quantitative analysis. Somewhat surprisingly, we did not detect some of the expected meiotic proteins, for example FANCM, HEI10, MLH3, RAD17, or RTEL1. Similarly, they were not identified in our previous proteomic study of early meiotic phase barley anthers (Lewandowska *et al.*, 2019). We recently showed that FANCM, HEI10, MLH3, RAD17, or RTEL1 genes are stably expressed but at a relatively low level in all stages of early meiotic anther development (Barakate *et al.*, 2021), suggesting that their protein products are also of low abundance and might be below the detection limit of the proteomics approach used in our study.

**Fig. 3.**
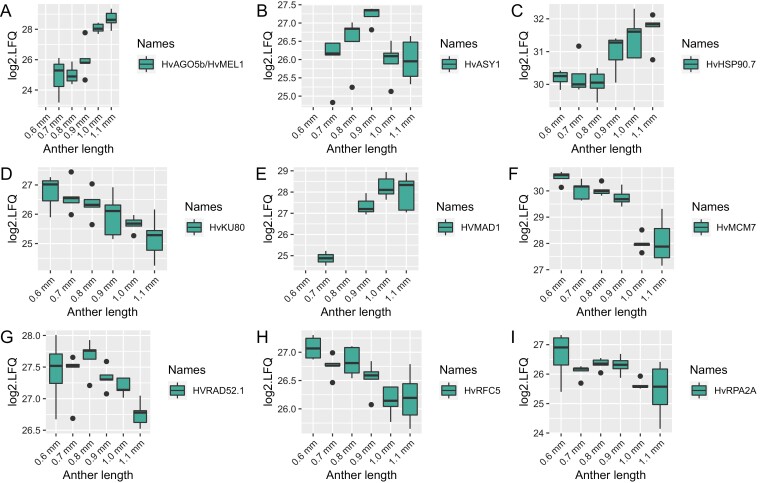
Differential protein abundance of nine significant meiosis-related proteins identified in BaAP. Individual points represent outliers.

We then inspected the group of 336 DAPs to check whether it included any known or putative meiotic proteins, as we may expect to see their abundance alter dynamically in the reproductive tissue. Nine putative meiotic proteins showed differential protein abundance in at least one stage comparison: HvASY1, HvAgo5b/MEL1, HvHSP90.7, HvKU80, HvMCM7, HvRPA2A, HvMAD1, HvRAD52.1, and HvRFC5 (Fig. 3). HvAGO5b/MEL1, HvHSP90.7. and HvMAD1 showed increased abundance in parallel to prophase I progression, while the abundances of HvKU80, HvMCM7, HvRPA2A, HvRAD52.1, and HvRFC5 exhibited the opposite trend, decreasing over the studied developmental timeline. The unique abundance pattern of HvASY1 corresponds to its behaviour in meiocytes shown by immunolocalization with anti-ASY1 antibodies (Fig. 1). In brief, HvASY1 was not detected in pre-meiotic anthers and its abundance increased until anthers reached zygotene at 0.9mm in length. When synapsis is completed in pachytene (anther 1.0mm), the abundance of HvASY1 starts to decrease.

### AGO protein abundance dynamically changes during meiosis I

**Fig. 4.**
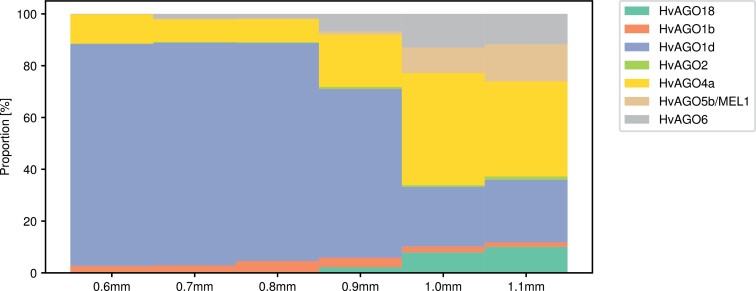
Anther size resolved the total protein intensity that is a mean intensity of the given protein in five replicates (four for sample 0.6mm in replicate 5) of the specific anther size sample and relative proportions of AGO proteins.

**Fig. 5.**
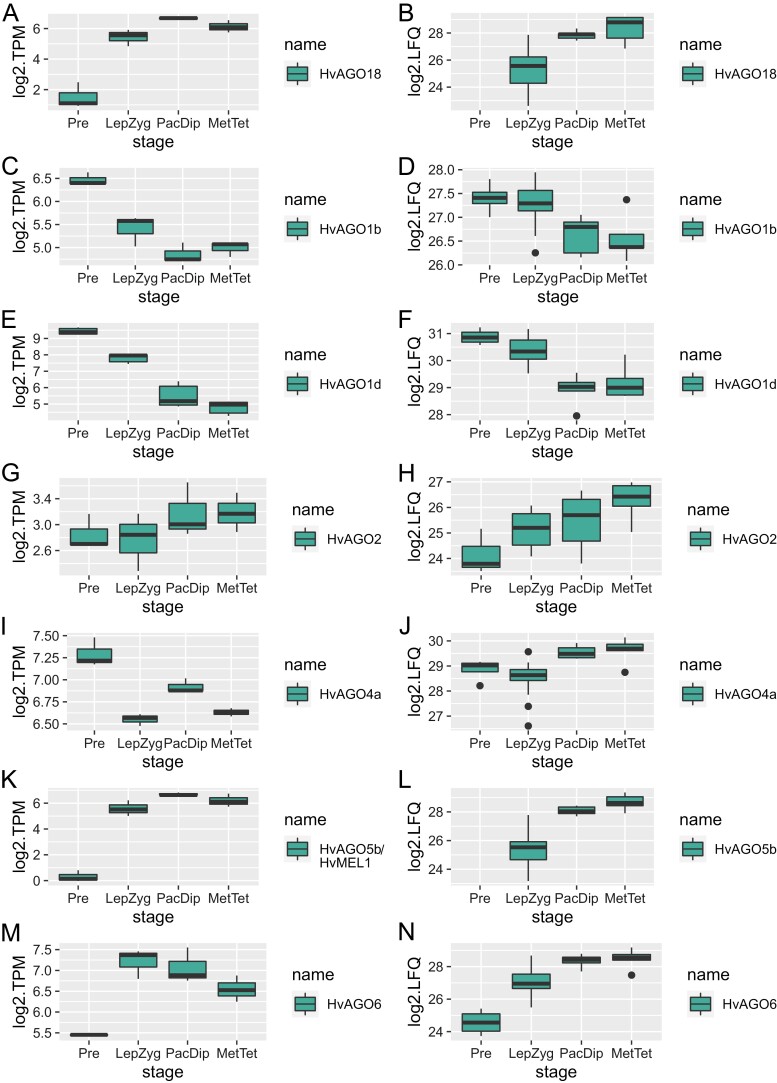
Comparison of the pattern of AGO gene expression in the barley anther transcriptome (BAnTr) (Barakate *et al.*, 2021) with BaAP AGO protein abundance. For box plots, BaAP anther lengths were binned according to their meiotic stage relative to the BAnTr dataset: 0.6mm, pre-meiotic; 0.7–0.9mm, leptotene–zygotene; 1.0mm, pachytene–diplotene; and 1.1mm, metaphase I. Individual points represent outliers.

We detected 13 out of 21 known plant AGO orthologues within BaAP: HvAGO18, HvAGO1b, HvAGO1c, HvAGO1d, HvAGO1e, HvAGO2, HvAGO3a, HvAGO3b, HvAGO4a, HvAGO4b, HvAGO5b/MEL1, HvAGO5c, and HvAGO6 (Table 2). Transcription of all 13 of these AGO genes was confirmed in our anther and meiocyte transcriptomics study (Barakate *et al.*, 2021). Notably, the abundance of seven of the identified AGO proteins (HvAGO18, HvAGO1b, HvAGO1d, HvAGO2, HvAGO4a, HvAGO5b/MEL1, and HvAGO6) significantly changed between different stages of our anther developmental timeline. Strikingly, various AGO proteins showed different and dynamic patterns of abundance in the developing anther, resulting in robust changes in their relative proportions (Fig. 4) and suggesting that individual AGO proteins have unique functions during specific substages of meiotic progression. For example, the abundance of AGO1d was continually down-regulated during anther development, while the majority of AGO proteins showed the opposite trend. The abundance of HvAGO18 and HvAGO5b/MEL1 was considerably different from that of the other five differentially abundant AGO proteins, highlighted by their absence in the pre-meiotic anthers (0.6mm). We have previously shown that the transcript levels of all seven AGO proteins revealed as differentially abundant here also changed, with differential expression of *HvAGO18*, *HvAGO1d*, *HvAGO2*, *HvAGO5b/MEL1*, and *HvAGO6* defined as statistically significant (Barakate *et al.*, 2021). Furthermore, transcripts of *HvAGO18*, *HvAGO2*, *HvAGO3a*, *HvAGO3b/MEL1*, *HvAGO5b*, and *HvAGO6* were highly enriched in the meiocytes compared with the whole anther, supporting their role in meiotic progression. Indeed, during anther development, the differential abundance of all AGO proteins (except HvAGO6) effectively paralleled their transcript abundance trend (Barakate *et al.*, 2021) (Fig. 5). Finally, within BaAP, we identified orthologues of other proteins involved in small RNAs biogenesis and gene silencing by RNA, with some also differentially abundant in the developing barley anther (Table 2). HvRDR6 (RNA-dependent RNA polymerase 6) (Harmoko *et al.*, 2013), HvIDN2 (Involved in De Novo 2) (Ausin *et al.*, 2012), HvKTF1 (Kow domain-containing transcription factor 1) (He *et al.*, 2009), and HvDRB4 (Double-stranded RNA-binding protein 4) (Chiliveri *et al.*, 2017) significantly changed in at least one stage comparison (Table 2).

**Table 2.** Orthologues of proteins involved in siRNA biogenesis/silencing by RNA identified in the BaAP

BaMP	Protein	DAP	NP	*Q*-value
BaMP.GP.3HG0667	HvAGO18	+	44	0
BaMP.GP.2HG2827	HvAGO1b	+	53	0
BaMP.GP.UnG0888	HvAGO1c		49	0
BaMP.GP.7HG3727	HvAGO1d	+	61	0
BaMP.GP.7HG0197	HvAGO1e		57	0
BaMP.GP.2HG2956	HvAGO2	+	36	0
BaMP.GP.2HG2955	HvAGO3a		17	0
BaMP.GP.2HG2952	HvAGO3b		9	0
BaMP.GP.3HG1239	HvAGO4a	+	56	0
BaMP.GP.1HG2953	HvAGO4b		56	0
BaMP.GP.2HG0912	HvAGO5b/MEL1	+	49	0
BaMP.GP.5HG3299	HvAGO5c		67	0
BaMP.GP.5HG1294	HvAGO6	+	50	0
BaMP.GP.5HG3366	HvCPSF100		22	0
BaMP.GP.3HG2990	HvDCL3		7	0
BaMP.GP.2HG2536	HvDCL4b		2	0.006589
BaMP.GP.1HG1043	HvDCL5		50	0
BaMP.GP.3HG2294	HvDRB4b	+	28	0
BaMP.GP.6HG1056	HvDRB5		2	0.000346
BaMP.GP.7HG3552	HvIDN2	+	20	0
BaMP.GP.1HG2268	HvKTF1	+	45	0
BaMP.GP.3HG3481	HvRDR6	+	46	0

DAP, differentially abundant protein (proteins identified as DAPs are marked with ‘+’); NP, number of matched peptides; *Q*-value, the ratio of reverse to forward protein groups generated by MaxQuant software. It operates as the *P*-value, where smaller is more significant and reflects better match quality.

### Dynamics of anther-specific proteins

To identify proteins involved in anther development within BaAP, we created a custom list of known and putative anther-specific genes based on published studies. The list consisted of genes encoding transcription factors (TFs) regulating tapetum development, including basic helix–loop–helix (bHLH) (Zhang *et al.*, 2006), MYB (Zhu *et al.*, 2008), and MADS (Hu *et al.*, 2011); a set of transcriptionally co-regulated meiotic-stage anther-specific *Arabidopsis thaliana* genes from Cluster 37 in FlowerNet (Pearce *et al.*, 2015); rice anther and pollen development genes from Clade 1.1 and Clade 1.2 in RiceAntherNet (Lin *et al.*, 2017); Triticale non-specific lipid transfer protein (LTP) genes; and genes with putative roles in pollen wall formation (Zaidi *et al.*, 2020). We then searched for and analysed the functionally annotated putative othologues of the genes on this list in our proteomic dataset. Next, we added BaAP proteins associated with GO terms connected with anther development: GO:0048658, GO:0048653, GO:0048655, GO:0048654, GO:0048657, GO:0048656, and GO:0010234. In total, we identified a robust set of 181 proteins with putative functions in anther development, and 14 of these showed differential protein abundance in at least one stage comparison (Supplementary Table S6). Twelve BaAP proteins with roles in sporopollenin synthesis, pollen wall formation, and anther dehiscence (Pearce *et al.*, 2015) were consistently up-regulated during anther development and growth from 0.6mm to 1.1mm. Surprisingly, among 26 TFs identified in BaAP, we did not detect any that were differentially abundant from the well-known anther and pollen development gene network comprising bHLH and MYB TFs (Sorensen *et al.*, 2003; Zhang *et al.*, 2006; Ito *et al.*, 2007; Zhang *et al.*, 2007; Zhu *et al.*, 2008). An orthologue of Aborted Microspores (AMS) was detected in our dataset, but its abundance did not significantly change in any stage comparison. TGAL7, a homologue of the *A. thaliana* TGA10 basic leucine zipper TF, was the only differentially abundant TF with predicted functions in anther development (Murmu *et al.*, 2010). It was down-regulated in several stage comparisons: 0.6mm with 1.1mm, 0.7mm with 1.1mm, and 0.8mm with 1.1mm (Supplementary Table S3). Another protein showing significant reduction in abundance across our developmental series was fatty acid hydroxylase domain-containing protein associated with GO term GO:0048658 ‘anther wall tapetum development’ (Supplementary Table S3).

It has been recently reported that AGO2 controls production of reactive oxygen species and the initiation of programmed cell death (PCD) of tapetum by epigenetic regulation of hexokinase 1 (HXK1) expression in rice anthers (Zheng *et al.*, 2019). HvAGO2 is significantly differentially expressed in our study and its abundance steadily increases in the developing anther (Fig. 5; Supplementary Table S3).

### Protein methylation in early developing meiotic-stage barley anthers

To check the methylation state of the BaAP, we searched MS/MS spectra of all 30 biological samples for peptides with arginine and lysine methylation. Considering the suboptimal amounts of starting material for the analysis, we could not apply any of the affinity purification techniques to enrich for methylated peptides. Nonetheless, in total, we identified 300 peptides with this modification: 84 arginine and 216 lysine methylated peptides, which correspond to 442 methylation sites on 197 proteins (Supplementary Table S7). The number of methylations in a single peptide varied between one, two, or three sites, and most of the proteins contained only one methylated peptide. Among detected arginine and/or lysine methylated proteins, there are some with potential functions in barley meiosis and anther development: HvAGO5b/MEL1, HvAGO1b, and HvAGO1e AGO proteins; five histones, and several putative meiotic proteins, namely HvRCC2, HvHOP2, HvFIP37, and HvHSP90.

## Discussion

A major goal of the current study was to acquire a global quantitative proteomic picture of developing anthers undergoing various stages of early meiosis in barley. To monitor protein abundance, we carried out high-resolution MS analysis of barley anthers, collected at six time points across an ~48h developmental window covering the entirety of prophase I. We used immunocytology to carefully stage each of the harvested samples and applied a previously published micro-proteomics workflow to carry out the proteomic analysis. We built a comprehensive Barley Anther Proteome (BaAP) by searching the MS data against a customized barley protein database (BaAP-Db) and identified 6431 non-redundant proteins (6105 identified by two or more peptides) in all 30 samples. We investigated how the proteome changed during early meiotic stage anther development, which represents a significant improvement of protein coverage over the existing literature.

The proteomes of developing anthers have rarely been explored, with only a few reports available (Kerim *et al.*, 2003; Ischebeck *et al.*, 2014; Yuan *et al.*, 2018; Lee *et al.*, 2020). Most of these considered a wide time scale of anther and pollen development and were not focused on the early meiotic stages. We therefore present the first quantitative comparative proteomics study of barley anther development during meiotic prophase I.

### Relative protein abundance is stable throughout prophase I

Within our dataset, we found that 336 proteins, ~5% of all identified proteins, changed in abundance significantly within our window of developmental time (Supplementary Table S3). This suggests that the meiotic phase barley anther proteome is relatively stable. Similar conclusions were reported by Ischebeck *et al.* (2014), who showed that the tobacco so-called ‘sporophytic proteome’, ranging from microsporocytes through meiocytes and tetrads up to microspores, is not very dynamic. It contained a high abundance of HSPs, RNA-binding proteins, and chromosome structure proteins (Ischebeck *et al.*, 2014). GO enrichment analysis of our BaAP revealed proteins associated with similar biological processes: ‘protein folding’, ‘response to heat’, ‘RNA processing’, ‘RNA transport’, and ‘chromatin organisation’ among others (Supplementary Table S2; Fig. 3).

We anticipate that the low number of detected DAPs in our study is possibly due to the temporary reduction of protein translation during meiosis and cell division, as described for mitosis (Sivan and Elroy-Stein, 2008), combined with the ‘dilution’ effect of sampling whole anthers on the sensitivity of our analyses. Protein synthesis is one of the most energy-demanding cellular processes and must be finely regulated. Our data suggest that in anthers during meiotic prophase I there is only a limited group of differentially abundant proteins. A total of 336 significant DAPs comprised proteins involved in biological processes such as DNA replication, double-strand break repair, and gene silencing by RNA (Supplementary Fig. S2B; Supplementary Table S3), all consistent with meiosis being the major dynamic process occurring in anthers at this developmental stage. The significance of meiosis-related mechanisms is further supported by our observation that transcripts encoding 65 of the 336 DAPs were preferentially expressed in meiocytes versus whole anthers during early meiosis (Barakate *et al.*, 2021). Other enriched biological processes included glycolysis and photosynthesis, emphasizing the requirements of the rapidly growing anther for both energy and nutrients.

While, to our knowledge, there is no quantitative proteomic study of early meiosis-stage anthers in a small grain cereal that we can compare our results with, several reports do describe developmental changes in the anther transcriptome (Chen *et al.*, 2010; Yang *et al.*, 2011; Dukowic-Schulze *et al.*, 2014; Barakate *et al.*, 2021). In the anthers of barley (Barakate *et al.*, 2021) and other species (Chen *et al.*, 2010; Yang *et al.*, 2011; Dukowic-Schulze *et al.*, 2014), a large-scale transcriptional switch occurs between pre-meiosis and leptotene. After this, in general terms, both meiocytes and whole anthers show a relatively stable pattern of transcript abundance at least until the late stages of prophase I (Barakate *et al.*, 2021). Stable protein abundance is also reflected in our proteomic data where only ~5% of the robustly detected anther proteins (336) showed evidence of differential abundance over developmental time. Approximately 4% of these DAPs (16) were putative meiotic proteins, which can be related to the small percentage of meiocytes relative to the bulk anther tissues. DAPs showing the highest LFC between comparisons of any two stages (log2 fold change >6) were mainly enzymes responsible for biosynthesis of fatty acids and protein glycosylation, reflecting the significant processes required for a rapidly growing anther, which almost doubles its size within the studied window of developmental time (48h).

The most significant changes when comparing adjacent stages in the anther proteome occurred during the transition from zygotene (0.9mm anther) to pachytene–diplotene (1mm anther) where 58 proteins showed differential abundance.

### Meiotic protein abundance and post-translational modifications during early meiosis

One of our main objectives was to identify and quantify proteins involved in meiotic prophase I using staged paired anthers and our micro-proteomics approach. Within the 6431 proteins identified, 82 were known or putative meiotic proteins, including some well-known players (e.g. HvASY1, HvDMC1, HvPCH2, HvTOPIIA, HvMEL1, and HvZYP1) (Table 1). On the other hand, we were not able to detect some key meiotic proteins, such as HvMER3 or HvSPO11-1. We assume it is a consequence of significant dilution of meiocytes by other anther cell types. Unsurprisingly, transcripts for all 82 identified meiotic proteins were detected previously in meiotic-phase anthers (Barakate *et al.*, 2021). Interestingly, 69 out of 82 proteins were detected in pre-meiotic anthers, suggesting that many of the proteins required to efficiently orchestrate meiosis have already been synthesized in advance of the onset of prophase I. Meiosis-associated proteins not identified in the pre-meiotic stage were HvASY1, HvAgo5b/MEL1, HvMAD1, HvMRE11A, HvMSH2, HvMSH6, HvNBS1, HvRPA2B, HvRPA2C, HvSMC1, HvSMG7, HvZIP1, and HvXRCC4 (Table 1). Notably, transcripts for all 13 genes have been detected in pre-meiotic anthers, however expression of HvAgo5b/MEL1, HvMSH6, HvRPA2C, and HvZIP1 was low (below 10 TPM) (Barakate *et al.*, 2021).

Similar findings were presented by Yuan *et al.* (2018) who detected 56 meiosis-related proteins in pre-meiotic maize anthers. One interpretation is that proteins are synthesized before they are required in preparation for their rapid functional activation that may be controlled at the post-translational level (Li *et al.*, 2014). Indeed, it has been clearly established that during meiosis, a range of protein modifications are commonly required for protein function. For example, protein SUMOylation, N-terminal acetylation, and phosphorylation regulate the assembly and disassembly of the synaptonemal complex, the structure that links homologous chromosomes during meiosis, and that temporal changes in this structure are linked to meiotic progression (Gao and Colaiácovo, 2018). Recent studies also point out the importance of protein methylation for meiosis progression. Histone H3 lysine 4 trimethylation (H3K4) is crucial for the formation of double-strand breaks in budding yeast (Borde *et al.*, 2009; Sommermeyer *et al.*, 2013) and for gametophyte development in plants (Berr *et al.*, 2010; Pinon *et al.*, 2017). Our preliminary analysis of barley proteome methylation detected arginine and lysine methylations in four histones, three AGO proteins (HvAGO5b/MEL1, HvAGO1b, and HvAGO1e), and meiotic-related proteins HvRCC2 and HvHSP90.7, suggesting that this type of post-translational modification is important for anther development. Investigating changes in protein modifications over developmental time could therefore be a fruitful but technically challenging addition to the work we describe here.

Despite the known prevalence of post-translational modifications, we expected a proportion of meiotic proteins to be differentially abundant throughout prophase I. Focusing on the 82 meiosis-related proteins detected in BaAP, only nine, HvASY1, HvAgo5b/MEL1, HvHSP90.7, HvKU80, HvMCM7, HvRPA2A, HvMAD1, HvRAD52.1, and HvRFC5, significantly changed in abundance across the six sampled stages of barley prophase I. We could observe two different trends in the abundance changes of meiotic DAPs: increasing (for HvAGO5b/MEL1, HvHSP90.7, and HvMAD1) or decreasing in parallel to prophase I progression (for HvKU80, HvMCM7, HvRPA2A, HvRAD52.1, and HvRFC5). Our findings agree with the reported roles of the meiotic DAPs. HvAGO5b/MEL1, HvHSP90.7, and HvMAD1 are all known to be involved in the later stages of meiotic prophase I. For example, the molecular chaperone HSP90 is required for synaptonemal complex disassembly and meiotic progression after pachytene in mice spermatocytes (Grad *et al.*, 2010) while rice MEL1 (MEIOSIS ARRESTED AT LEPTOTENE1) is indispensable for meiosis progression (Nonomura *et al.*, 2007) and is degraded after meiosis (Lian *et al.*, 2021). The spindle assembly checkpoint component MAD1 is essential for synapsis and accurate segregation of chromosomes during meiosis (Gorbsky, 2015). Meiosis-related DAPs showing a decreasing abundance in our dataset function in the early meiosis stages so their lower abundance towards the end of prophase I is expected. The main role of Replication Protein A2A (RPA2A), DNA replication licensing factor MCM7, and Replication Factor C5 (RFC) is the DNA replication preceding meiosis I (Elmayan *et al.*, 2005; Evrin *et al.*, 2009; Xia *et al.*, 2010) while RADiation-sensitive52 (RAD52) initiates homologous recombination (Samach *et al.*, 2011) and ATP-dependent DNA helicase 2 subunit KU80 is involved in the non-homologous DNA end joining (NHEJ) necessary for double-strand break repair (West *et al.*, 2002). HvASY1 abundance in our experiment was unique (Figs 1, 3B) and consistent with the published studies showing high ASY1 dynamics and implication in multiple meiotic processes including synapsis, axis morphogenesis, and coordinating the activity of other members of the homologous recombination machinery (Armstrong *et al.*, 2002; Nonomura *et al.*, 2004; Sanchez-Moran *et al.*, 2007; Cuacos *et al.*, 2021).

In Barakate *et al.* (2021), 29 differentially expressed putative meiotic genes were detected across prophase I but only three of them were identified as DAPs in our study, namely HvASY1, HvAgo5b/MEL1, and HvHSP90.7. We assume the lower number of DAPs may largely be a consequence of both the lower resolution of the proteomic versus transcriptomic approach and the fine-tuning of meiotic gene expression at translation and post-translational levels.

### Small RNA biogenesis pathway proteins are dynamic and abundant in meiotic prophase I

Recent studies suggest that anther-specific small RNAs, including miRNAs and phasiRNAs, can alter meiotic gene expression and the distribution of double-strand breaks (Huang *et al.*, 2019). Moreover, the RNA-directed DNA methylation (RDDM) pathway is emerging as a new mechanism of meiotic regulation and has been shown to induce cell lineage-specific epigenetic marks regulating meiotic gene expression in Arabidopsis (Walker *et al.*, 2018).

The GO term ‘gene silencing by RNA’ was one of the top enriched terms identified by GO enrichment analysis of the 336 DAPs, indicating the importance of this pathway for barley anther development. In the BaAP, we detected 22 proteins involved in the biogenesis of small RNAs including 13 AGO proteins, and half of them (11) were classified as differentially abundant. (Table 2)

Exploring the abundance of identified AGO proteins, we found that seven changed significantly during anther development: HvAGO18, HvAGO1b, HvAGO1d, HvAGO2, HvAGO4a, HvAGO5b/MEL1, and HvAGO6. Functions of all seven AGO DAPs in male reproductive organ development and/or meiosis progression are well documented (Bélanger *et al.*, 2020; Zhang *et al.*, 2020; Lian *et al.*, 2021).

As expected, most of the abundance profiles of AGO proteins observed during early anther development in this study, mirrored their gene expression patterns reported by Barakate *et al.* (2021). A slight discrepancy between transcript and protein abundance was however noted for HvAGO6 and HvAGO4a, pinpointing the importance of post-transcriptional processes in regulation of their expression. Both proteins belong to the modifier AGO clade, members of which can bind 24 nt siRNAs as well as 21 nt tasiRNAs, both involved in the RDDM pathway (Wu *et al.*, 2012; Borges and Martienssen, 2015). Moreover, AGO4 was reported to be required for accurate chromosome segregation during anaphase I (Oliver *et al.*, 2016).

An interesting finding from our BaAP methylome study was the identification of mono- and dimethylated arginine residues in three AGO proteins, HvAGO5b/MEL1, HvAGO1b, and HvAGO1e. Nguyen and Phillips (2020) described the *Caenorhabditis elegans* CSR-1 AGO protein isoform that contains multiple dimethylarginine modifications that are crucial for the preferential binding to spermatogenesis-specific small RNAs. This suggests that methylation might be a mechanism for individual AGO proteins to obtain small RNA specificity.

Further functional analyses will be needed to fully understand the role of specific AGO proteins and corresponding small RNAs in cereal meiosis.

### Anther-specific proteins

As our developmental time series consisted of the whole barley anthers, we had the opportunity to investigate the simultaneous dynamics of the proteome in both gametophytic and sporophytic tissues. We catalogued a collection of 181 previously characterized proteins considered to be involved in anther and tapetum development in our dataset (Supplementary Table S6). Among these, we identified proteins involved in the early stages of anther development including leucine-rich repeat receptor protein kinases (LRR-RLKs), an orthologue of rice Multiple Sporocyte 1 (MSP1), and two orthologues of Barely Any Meristem 1 (BAM). Nonomura *et al.* (2003) reported that rice mutants with defective MSP1 had impaired tapetum layer initiation and male meiosis aborted before the end of prophase I. Arabidopsis BAM1 is involved in cell division, differentiation, and sporophytic anther tissue specification (Hord *et al.*, 2006).

In the barley anther proteome, we identified 26 TFs with predicted functions in tapetum development, including bHLHs (Zhang *et al.*, 2006), MYBs (Zhu *et al.*, 2008), and MADS (Hu *et al.*, 2011). TFs known to act at the early stages of anther development included a bHLH protein, TDR Interacting Protein2 (TIP2) (Fu *et al.*, 2014), which coordinates the initiation and development of the tapetum in rice by modulating the transcription of Tapetum Degeneration Retardation (TDR), a protein also identified in BaAP (Li *et al.*, 2006). We did not observe any significant changes in the abundance of any TFs, with one exception, the basic leucine zipper transcription factor TGAL7/TGA9, reported to be indispensable for the stability of the middle layer and degeneration of endothecium and epidermis and, consequently, for pollen fertility (Murmu *et al.*, 2010). We observed a decreasing abundance of TGAL7/TGA9 in the more meiotically advanced anthers.

In conclusion, we have generated the most comprehensive proteome information so far using a protein database tailored to the cultivar and experiment using transcriptomics data, and detected >6100 proteins in meiotically staged paired barley anthers that, on global analysis, broadly represents the classes of proteins expected of a rapidly developing tissue containing a subset of differentiating gametophytic cells. We identified 336 of these proteins as robustly differentially abundant. Within this subset, we identified several proteins both in the whole anther and, more specifically, in meiocytes that are worthy of further investigation. For example, given our primary interest in meiosis, we were particularly intrigued by the family of differentially abundant AGO proteins, especially as our previous transcriptomics data support their enrichment in meiocytes. The overall stability of protein abundance was also a major feature of our data. We are mindful that our approach was not able to capture information on many important post-translational modifications other than methylation that from previously published work we expect may be essential to progression of meiotic prophase I (Dong and Han, 2012; Ye et al., 2015, 2016; Li *et al.*, 2018). Consequently, our future proteomic studies will focus on isolated meiocytes/meiocyte sacs and sensitive approaches to the detection of protein modifications, while also exploring some of the identified differentially abundant meiotic proteins at the functional level.

## Supplementary data

The following supplementary data are available at *JXB* online.

Fig. S1. Percentage of BaAP proteins identified with one, more than two, and more than 10 peptides.

Fig. S2. GO enrichment analysis of all BaAP proteins (A), all differentially abundant proteins (DAPs) (B), and DAPs identified in comparison between stages 0.9mm and 1.0mm (C).

Fig. S3. Sample clustering by principal component analysis (PCA) by anther stage.

Table S1. All identified BaAP proteins with PANNZER and EggNOG GO annotations.

Table S2. GO enrichment analysis of all identified BaAP proteins.

Table S3. List of 336 BaAP proteins determined to be DAPs and DAP GO enrichment analysis.

Table S4. List of DAPs observed when comparing the proteome of 0.6mm anthers versus 1.1mm anthers.

Table S5. List of DAPs observed when comparing the proteome of 0.9mm anthers versus 1.0mm anthers.

Table S6. List of BaAP proteins with known or putative functions in anther and tapetum development.

Table S7. List of methylated peptides identified in BaAP.

erab494_suppl_Supplementary_Figures_S1-S3

erab494_suppl_Supplementary_Table_S1

erab494_suppl_Supplementary_Table_S2

erab494_suppl_Supplementary_Table_S3

erab494_suppl_Supplementary_Table_S4

erab494_suppl_Supplementary_Table_S5

erab494_suppl_Supplementary_Table_S6

erab494_suppl_Supplementary_Table_S7

## Data Availability

The mass spectrometry proteomics data have been deposited to the ProteomeXchange Consortium via the PRIDE (Perez-Riverol *et al.*, 2019) partner repository with the dataset identifier PXD026200.

## Abbreviations

BAnTr: Barley Anther Transcriptome
BaP: Barley Anther Proteome
DAP: differentially abundant protein
TF: transcription factor

## References

[CIT0001] Alexa A , Rahnenführer J, Lengauer T. 2006. Improved scoring of functional groups from gene expression data by decorrelating GO graph structure. Bioinformatics 22, 1600–1607. 16606683 10.1093/bioinformatics/btl140

[CIT0002] Alexa A , Rahnenfuhrer J. 2020. topGO: enrichment analysis for Gene Ontology. R package version 2.46.0.

[CIT0003] Armstrong SJ , Caryl AP, Jones GH, Franklin FC. 2002. Asy1, a protein required for meiotic chromosome synapsis, localizes to axis-associated chromatin in Arabidopsis and Brassica. Journal of Cell Science 115, 3645–3655. 12186950 10.1242/jcs.00048

[CIT0004] Arrieta M , Colas I, Macaulay M, Waugh R, Ramsay L. 2020. A modular tray growth system for barley, in plant meiosis. Methods in Molecular Biology 2061, 367–379. 31583673 10.1007/978-1-4939-9818-0_26

[CIT0005] Ausin I , Greenberg MV, Simanshu DK, et al. 2012. INVOLVED IN DE NOVO 2-containing complex involved in RNA-directed DNA methylation in Arabidopsis. Proceedings of the National Academy of Sciences, USA 109, 8374–8381. 10.1073/pnas.1206638109 PMC3365198 22592791

[CIT0006] Barakate A , Orr J, Schreiber M, et al. 2021. Barley anther and meiocyte transcriptome dynamics in meiotic prophase I. Frontiers in Plant Science 11, 619404. 33510760 10.3389/fpls.2020.619404 PMC7835676

[CIT0007] Bélanger S , Pokhrel S, Czymmek K, Meyers BC. 2020. Premeiotic, 24-nucleotide reproductive PhasiRNAs are abundant in anthers of wheat and barley but not rice and maize. Plant Physiology 184, 1407–1423. 32917771 10.1104/pp.20.00816 PMC7608162

[CIT0008] Berr A , McCallum EJ, Ménard R, Meyer D, Fuchs J, Dong A, Shen WH. 2010. Arabidopsis SET DOMAIN GROUP2 is required for H3K4 trimethylation and is crucial for both sporophyte and gametophyte development. The Plant Cell 22, 3232–3248. 21037105 10.1105/tpc.110.079962 PMC2990135

[CIT0009] Borde V , Robine N, Lin W, Bonfils S, Géli V, Nicolas A. 2009. Histone H3 lysine 4 trimethylation marks meiotic recombination initiation sites. The EMBO Journal 28, 99–111. 19078966 10.1038/emboj.2008.257 PMC2634730

[CIT0010] Borges F , Martienssen RA. 2015. The expanding world of small RNAs in plants. Nature Reviews. Molecular Cell Biology 16, 727–741. 26530390 10.1038/nrm4085 PMC4948178

[CIT0011] Chen C , Farmer AD, Langley RJ, Mudge J, Crow JA, May GD, Huntley J, Smith AG, Retzel EF. 2010. Meiosis-specific gene discovery in plants: RNA-Seq applied to isolated Arabidopsis male meiocytes. BMC Plant Biology 10, 280. 21167045 10.1186/1471-2229-10-280 PMC3018465

[CIT0012] Chen Z , Zhong W, Chen S, Zhou Y, Ji P, Gong Y, Yang Z, Mao Z, Zhang C, Mu F. 2021. TMT-based quantitative proteomics analyses of sterile/fertile anthers from a genic male-sterile line and its maintainer in cotton (*Gossypium hirsutum* L.). Journal of Proteomics 232, 104026. 33127528 10.1016/j.jprot.2020.104026

[CIT0013] Chiliveri SC , Aute R, Rai U, Deshmukh MV. 2017. DRB4 dsRBD1 drives dsRNA recognition in *Arabidopsis thaliana* tasi/siRNA pathway. Nucleic Acids Research 45, 8551–8563. 28575480 10.1093/nar/gkx481 PMC5737894

[CIT0014] Colas I , Darrier B, Arrieta M, Mittmann SU, Ramsay L, Sourdille P, Waugh R. 2017. Observation of extensive chromosome axis remodeling during the ‘diffuse-phase’ of meiosis in large genome cereals. Frontiers in Plant Science 8, 1235. 28751906 10.3389/fpls.2017.01235 PMC5508023

[CIT0015] Collado-Romero M , Alós E, Prieto P. 2014. Unravelling the proteomic profile of rice meiocytes during early meiosis. Frontiers in Plant Science 5, 356. 25104955 10.3389/fpls.2014.00356 PMC4109522

[CIT0016] Cox J , Mann M. 2008. MaxQuant enables high peptide identification rates, individualized p.p.b.-range mass accuracies and proteome-wide protein quantification. Nature Biotechnology 26, 1367–1372. 10.1038/nbt.1511 19029910

[CIT0017] Cox J , Neuhauser N, Michalski A, Scheltema RA, Olsen JV, Mann M. 2011. Andromeda: a peptide search engine integrated into the MaxQuant environment. Journal of Proteome Research 10, 1794–1805. 21254760 10.1021/pr101065j

[CIT0018] Cuacos M , Lambing C, Pachon-Penalba M, Osman K, Armstrong SJ, Henderson IR, Sanchez-Moran E, Franklin FCH, Heckmann S. 2021. Meiotic chromosome axis remodelling is critical for meiotic recombination in *Brassica rapa*. Journal of Experimental Botany 72, 3012–3027. 33502451 10.1093/jxb/erab035 PMC8023211

[CIT0019] Dong Q , Han F. 2012. Phosphorylation of histone H2A is associated with centromere function and maintenance in meiosis. The Plant Journal 71, 800–809. 22519817 10.1111/j.1365-313X.2012.05029.x

[CIT0020] Dukowic-Schulze S , Sundararajan A, Mudge J, et al. 2014. The transcriptome landscape of early maize meiosis. BMC Plant Biology 14, 118. 24885405 10.1186/1471-2229-14-118 PMC4032173

[CIT0021] Dukowic-Schulze S , Sundararajan A, Ramaraj T, Kianian S, Pawlowski WP, Mudge J, Chen C. 2016. Novel meiotic miRNAs and indications for a role of phasiRNAs in meiosis. Frontiers in Plant Science 7, 762. 27313591 10.3389/fpls.2016.00762 PMC4889585

[CIT0022] Elmayan T , Proux F, Vaucheret H. 2005. Arabidopsis RPA2: a genetic link among transcriptional gene silencing, DNA repair, and DNA replication. Current Biology 15, 1919–1925. 16271868 10.1016/j.cub.2005.09.044

[CIT0023] Evrin C , Clarke P, Zech J, Lurz R, Sun J, Uhle S, Li H, Stillman B, Speck C. 2009. A double-hexameric MCM2-7 complex is loaded onto origin DNA during licensing of eukaryotic DNA replication. Proceedings of the National Academy of Sciences, USA 106, 20240–20245. 10.1073/pnas.0911500106 PMC2787165 19910535

[CIT0024] Fei Q , Yang L, Liang W, Zhang D, Meyers BC. 2016. Dynamic changes of small RNAs in rice spikelet development reveal specialized reproductive phasiRNA pathways. Journal of Experimental Botany 67, 6037–6049. 27702997 10.1093/jxb/erw361 PMC5100018

[CIT0025] Feng N , Song G, Guan J, et al. 2017. Transcriptome profiling of wheat inflorescence development from spikelet initiation to floral patterning identified stage-specific regulatory genes. Plant Physiology 174, 1779–1794. 28515146 10.1104/pp.17.00310 PMC5490901

[CIT0026] Flórez-Zapata NMV , Reyes-Valdés MH, Hernandez-Godínez F, Martínez O. 2014. Transcriptomic landscape of prophase I sunflower male meiocytes. Frontiers in Plant Scicience 5, 277. 10.3389/fpls.2014.00277 PMC4059168 24982667

[CIT0027] Fu Z , Yu J, Cheng X, Zong X, Xu J, Chen M, Li Z, Zhang D, Liang W. 2014. The rice basic helix–loop–helix transcription factor TDR INTERACTING PROTEIN2 is a central switch in early anther development. The Plant Cell 26, 1512–1524. 24755456 10.1105/tpc.114.123745 PMC4036568

[CIT0028] Gao J , Colaiácovo MP. 2018. Zipping and unzipping: protein modifications regulating synaptonemal complex dynamics. Trends in Genetics 34, 232–245. 29290403 10.1016/j.tig.2017.12.001 PMC5834363

[CIT0029] Gierlinski M , Gastaldello F, Cole C, Barton G. 2018. Proteus: an R package for downstream analysis of MaxQuant output. BioRxiv. doi: 10.1101/416511 [Preprint].

[CIT0030] Goldberg RB , Beals TP, Sanders PM. 1993. Anther development: basic principles and practical applications. The Plant Cell 5, 1217–1229. 8281038 10.1105/tpc.5.10.1217 PMC160355

[CIT0031] Gómez JF , Talle B, Wilson ZA. 2015. Anther and pollen development: a conserved developmental pathway. Journal of Integrative Plant Biology 57, 876–891. 26310290 10.1111/jipb.12425 PMC4794635

[CIT0032] Gorbsky GJ. 2015. The spindle checkpoint and chromosome segregation in meiosis. The FEBS Journal 282, 2471–2487. 25470754 10.1111/febs.13166 PMC4454629

[CIT0033] Grad I , Cederroth CR, Walicki J, Grey C, Barluenga S, Winssinger N, De Massy B, Nef S, Picard D. 2010. The molecular chaperone Hsp90α is required for meiotic progression of spermatocytes beyond pachytene in the mouse. PLoS One 5, e15770. 21209834 10.1371/journal.pone.0015770 PMC3013136

[CIT0034] Harmoko R , Fanata WI, Yoo JY, et al. 2013. RNA-dependent RNA polymerase 6 is required for efficient hpRNA-induced gene silencing in plants. Molecules and Cells 35, 202–209. 23456296 10.1007/s10059-013-2203-2 PMC3887914

[CIT0035] Hatkevich T , Miller DE, Turcotte CA, Miller MC, Sekelsky J. 2021. A pathway for error-free non-homologous end joining of resected meiotic double-strand breaks. Nucleic Acids Research 49, 879–890. 33406239 10.1093/nar/gkaa1205 PMC7826270

[CIT0036] He XJ , Hsu YF, Zhu S, Wierzbicki AT, Pontes O, Pikaard CS, Liu HL, Wang CS, Jin H, Zhu JK. 2009. An effector of RNA-directed DNA methylation in arabidopsis is an ARGONAUTE 4- and RNA-binding protein. Cell 137, 498–508. 19410546 10.1016/j.cell.2009.04.028 PMC2700824

[CIT0037] Higgins JD , Perry RM, Barakate A, Ramsay L, Waugh R, Halpin C, Armstrong SJ, Franklin FC. 2012. Spatiotemporal asymmetry of the meiotic program underlies the predominantly distal distribution of meiotic crossovers in barley. The Plant Cell 24, 4096–4109. 23104831 10.1105/tpc.112.102483 PMC3517238

[CIT0038] Hord CL , Chen C, Deyoung BJ, Clark SE, Ma H. 2006. The BAM1/BAM2 receptor-like kinases are important regulators of Arabidopsis early anther development. The Plant Cell 18, 1667–1680. 16751349 10.1105/tpc.105.036871 PMC1488923

[CIT0039] Hu L , Liang W, Yin C, Cui X, Zong J, Wang X, Hu J, Zhang D. 2011. Rice MADS3 regulates ROS homeostasis during late anther development. The Plant Cell 23, 515–533. 21297036 10.1105/tpc.110.074369 PMC3077785

[CIT0040] Huang J , Wang C, Wang H, Lu P, Zheng B, Ma H, Copenhaver GP, Wang Y. 2019. Meiocyte-specific and AtSPO11-1-dependent small RNAs and their association with meiotic gene expression and recombination. The Plant Cell 31, 444–464. 30674694 10.1105/tpc.18.00511 PMC6447014

[CIT0041] Huybrechts M , Cuypers A, Deckers J, Iven V, Vandionant S, Jozefczak M, Hendrix S. 2019. Cadmium and plant development: an agony from seed to seed. International Journal of Molecular Sciences 20, 3971. 10.3390/ijms20163971 PMC6718997 31443183

[CIT0042] Ischebeck T , Valledor L, Lyon D, Gingl S, Nagler M, Meijón M, Egelhofer V, Weckwerth W. 2014. Comprehensive cell-specific protein analysis in early and late pollen development from diploid microsporocytes to pollen tube growth. Molecular & Cellular Proteomics 13, 295–310. 24078888 10.1074/mcp.M113.028100 PMC3879621

[CIT0043] Ito T , Nagata N, Yoshiba Y, Ohme-Takagi M, Ma H, Shinozaki K. 2007. Arabidopsis MALE STERILITY1 encodes a PHD-type transcription factor and regulates pollen and tapetum development. The Plant Cell 19, 3549–3562. 18032630 10.1105/tpc.107.054536 PMC2174881

[CIT0044] Kelliher T , Egger RL, Zhang H, Walbot V. 2014. Unresolved issues in pre-meiotic anther development. Frontiers in Plant Science 5, 347. 25101101 10.3389/fpls.2014.00347 PMC4104404

[CIT0045] Kerim T , Imin N, Weinman JJ, Rolfe BG. 2003. Proteome analysis of male gametophyte development in rice anthers. Proteomics 3, 738–751. 12748952 10.1002/pmic.200300424

[CIT0046] Kim M , Kim H, Lee W, Lee Y, Kwon SW, Lee J. 2015. Quantitative shotgun proteomics analysis of rice anther proteins after exposure to high temperature. International Journal of Genomics 2015, 238704. 26618163 10.1155/2015/238704 PMC4651674

[CIT0047] Knoll A , Puchta H. 2011. The role of DNA helicases and their interaction partners in genome stability and meiotic recombination in plants. Journal of Experimental Botany 62, 1565–1579. 21081662 10.1093/jxb/erq357

[CIT0048] Komiya R , Ohyanagi H, Niihama M, Watanabe T, Nakano M, Kurata N, Nonomura K. 2014. Rice germline-specific Argonaute MEL1 protein binds to phasiRNAs generated from more than 700 lincRNAs. The Plant Journal 78, 385–397. 24635777 10.1111/tpj.12483

[CIT0049] Lee Y , Kwon Y, Kim, J, et al. 2020. Monitoring rice anther proteome expression patterns during pollen development. Plant Biotechnology Reports 14, 293–300.

[CIT0050] Lewandowska D , Zhang R, Colas I, Uzrek N, Waugh R. 2019. Application of a sensitive and reproducible label-free proteomic approach to explore the proteome of individual meiotic-phase barley anthers. Frontiers in Plant Science 10, 393. 31001307 10.3389/fpls.2019.00393 PMC6454111

[CIT0051] Li GW , Burkhardt D, Gross C, Weissman JS. 2014. Quantifying absolute protein synthesis rates reveals principles underlying allocation of cellular resources. Cell 157, 624–635. 24766808 10.1016/j.cell.2014.02.033 PMC4006352

[CIT0052] Li N , Zhang DS, Liu HS, et al. 2006. The rice tapetum degeneration retardation gene is required for tapetum degradation and anther development. The Plant Cell 18, 2999–3014. 17138695 10.1105/tpc.106.044107 PMC1693939

[CIT0053] Li X , Ye J, Ma H, Lu P. 2018. Proteomic analysis of lysine acetylation provides strong evidence for involvement of acetylated proteins in plant meiosis and tapetum function. The Plant Journal 93, 142–154. 29124795 10.1111/tpj.13766

[CIT0054] Lian JP , Yang YW, He RR, et al. 2021. Ubiquitin-dependent Argonaute protein MEL1 degradation is essential for rice sporogenesis and phasiRNA target regulation. The Plant Cell 33, 2685–2700. 34003932 10.1093/plcell/koab138 PMC8408455

[CIT0055] Lin H , Yu J, Pearce SP, Zhang D, Wilson ZA. 2017. RiceAntherNet: a gene co-expression network for identifying anther and pollen development genes. The Plant Journal 92, 1076–1091. 29031031 10.1111/tpj.13744

[CIT0056] Ma H. 2005. Molecular genetic analyses of microsporogenesis and microgametogenesis in flowering plants. Annual Review of Plant Biology 56, 393–434. 10.1146/annurev.arplant.55.031903.141717 15862102

[CIT0057] Mackenzie G , Boa AN, Diego-Taboada A, Atkin SL, Sathyapalan T. 2015. Sporopollenin, the least known yet toughest natural biopolymer. Frontiers in Materials 2, 66.

[CIT0058] Martinez-Garcia M , Schubert V, Osman K, Darbyshire A, Sanchez-Moran E, Franklin FCH. 2018. TOPII and chromosome movement help remove interlocks between entangled chromosomes during meiosis. Journal of Cell Biology 217, 4070–4079. 10.1083/jcb.201803019 PMC6279386 30266762

[CIT0059] Murmu J , Bush MJ, DeLong C, Li S, Xu M, Khan M, Malcolmson C, Fobert PR, Zachgo S, Hepworth SR. 2010. Arabidopsis basic leucine-zipper transcription factors TGA9 and TGA10 interact with floral glutaredoxins ROXY1 and ROXY2 and are redundantly required for anther development. Plant Physiology 154, 1492–1504. 20805327 10.1104/pp.110.159111 PMC2971623

[CIT0060] Nguyen DAH , Phillips CM. 2020. Arginine methylation promotes siRNA-binding specificity for a spermatogenesis-specific isoform of the Argonaute protein CSR-1. Nature Communications 12, 4212. 10.1038/s41467-021-24526-6 PMC8270938 34244496

[CIT0061] Nonomura K , Miyoshi K, Eiguchi M, Suzuki T, Miyao A, Hirochika H, Kurata N. 2003. The MSP1 gene is necessary to restrict the number of cells entering into male and female sporogenesis and to initiate anther wall formation in rice. The Plant Cell 15, 1728–1739. 12897248 10.1105/tpc.012401 PMC167165

[CIT0062] Nonomura K , Morohoshi A, Nakano M, Eiguchi M, Miyao A, Hirochika H, Kurata N. 2007. A germ cell specific gene of the ARGONAUTE family is essential for the progression of premeiotic mitosis and meiosis during sporogenesis in rice. The Plant Cell 19, 2583–2594. 17675402 10.1105/tpc.107.053199 PMC2002623

[CIT0063] Nonomura KI , Nakano M, Murata K, Miyoshi K, Eiguchi M, Miyao A, Hirochika H, Kurata N. 2004. An insertional mutation in the rice PAIR2 gene, the ortholog of Arabidopsis ASY1, results in a defect in homologous chromosome pairing during meiosis. Molecular Genetics and Genomics 271, 121–129. 14758540 10.1007/s00438-003-0934-z

[CIT0064] Oliver C , Santos JL, Pradillo M. 2016. Accurate chromosome segregation at first meiotic division requires AGO4, a protein involved in RNA-dependent DNA methylation in *Arabidopsis thaliana*. Genetics 204, 543–553. 27466226 10.1534/genetics.116.189217 PMC5068845

[CIT0065] Ortega MA , Marh J, Alarcon VB, Ward WS. 2012. Unique pattern of ORC2 and MCM7 localization during DNA replication licensing in the mouse zygote. Biology of Reproduction 87, 62. 22674395 10.1095/biolreprod.112.101774 PMC3463414

[CIT0066] Parish RW , Li SF. 2010. Death of a tapetum: a programme of developmental altruism. Plant Science 178, 73–89.

[CIT0067] Pearce S , Ferguson A, King J, Wilson ZA. 2015. FlowerNet: a gene expression correlation network for anther and pollen development. Plant Physiology 167, 1717–1730. 25667314 10.1104/pp.114.253807 PMC4378160

[CIT0068] Perez-Riverol Y , Csordas A, Bai J, et al. 2019. The PRIDE database and related tools and resources in 2019: improving support for quantification data. Nucleic Acids Research 47, D442–D450. 30395289 10.1093/nar/gky1106 PMC6323896

[CIT0069] Pinon V , Yao X, Dong A, Shen WH. 2017. SDG2-mediated H3K4me3 is crucial for chromatin condensation and mitotic division during male gametogenesis in Arabidopsis. Plant Physiology 174, 1205–1215. 28455402 10.1104/pp.17.00306 PMC5462044

[CIT0070] Ruszkowski M , Sekula B, Ruszkowska A, Dauter Z. 2018. Chloroplastic serine hydroxymethyltransferase from *Medicago truncatula*: a structural characterization. Frontiers in Plant Science 9, 584. 29868052 10.3389/fpls.2018.00584 PMC5958214

[CIT0071] Sabi R , Tuller T. 2019. Novel insights into gene expression regulation during meiosis revealed by translation elongation dynamics. NPJ Systems Biology and Applications 5, 12. 30962948 10.1038/s41540-019-0089-0 PMC6449359

[CIT0072] Samach A , Melamed-Bessudo C, Avivi-Ragolski N, Pietrokovski S, Levy AA. 2011. Identification of plant RAD52 homologs and characterization of the *Arabidopsis thaliana* RAD52-like genes. The Plant Cell 23, 4266–4279. 22202891 10.1105/tpc.111.091744 PMC3269865

[CIT0073] Sanchez-Moran E , Santos JL, Jones GH, Franklin FC. 2007. ASY1 mediates AtDMC1-dependent interhomolog recombination during meiosis in Arabidopsis. Genes & Development 21, 2220–2233. 17785529 10.1101/gad.439007 PMC1950860

[CIT0074] Sivan G , Elroy-Stein O. 2008. Regulation of mRNA translation during cellular division. Cell Cycle 7, 741–744. 18239464 10.4161/cc.7.6.5596

[CIT0075] Sommermeyer V , Béneut C, Chaplais E, Serrentino ME, Borde V. 2013. Spp1, a member of the Set1 complex, promotes meiotic DSB formation in promoters by tethering histone H3K4 methylation sites to chromosome axes. Molecular Cell 49, 43–54. 23246437 10.1016/j.molcel.2012.11.008

[CIT0076] Sonneville R , Querenet M, Craig A, Gartner A, Blow JJ. 2012. The dynamics of replication licensing in live *Caenorhabditis elegans* embryos. Journal of Cell Biology 196, 233–246. 10.1083/jcb.201110080 PMC3265957 22249291

[CIT0077] Sorensen AM , Kröber S, Unte US, Huijser P, Dekker K, Saedler H. 2003. The Arabidopsis ABORTED MICROSPORES (AMS) gene encodes a MYC class transcription factor. The Plant Journal 33, 413–423. 12535353 10.1046/j.1365-313x.2003.01644.x

[CIT0078] Törönen P , Medlar A, Holm L. 2018. PANNZER2: a rapid functional annotation web server. Nucleic Acids Research 46, W84–W88. 29741643 10.1093/nar/gky350 PMC6031051

[CIT0079] Walker J , Gao H, Zhang J, Aldridge B, Vickers M, Higgins JD, Feng X. 2018. Sexual-lineage-specific DNA methylation regulates meiosis in Arabidopsis. Nature Genetics 50, 130–137. 29255257 10.1038/s41588-017-0008-5 PMC7611288

[CIT0080] Wang A , Xia Q, Xie W, Datla R, Selvaraj G. 2003. The classical Ubisch bodies carry a sporophytically produced structural protein (RAFTIN) that is essential for pollen development. Proceedings of the National Academy of Sciences, USA 100, 14487–14492. 10.1073/pnas.2231254100 PMC283618 14612572

[CIT0081] West CE , Waterworth WM, Story GW, Sunderland PA, Jiang Q, Bray CM. 2002. Disruption of the Arabidopsis AtKu80 gene demonstrates an essential role for AtKu80 protein in efficient repair of DNA double-strand breaks in vivo. The Plant Journal 31, 517–528. 12182708 10.1046/j.1365-313x.2002.01370.x

[CIT0082] Wickham H. 2016. ggplot2: elegant graphics for data analysis. New York, NY: Springer-Verlag.

[CIT0083] Wickham H , Henry L. 2020. tidyr: tidy messy data. https://CRAN.R-project.org/package=tidyr

[CIT0084] Wu L , Mao L, Qi Y. 2012. Roles of dicer-like and argonaute proteins in TAS-derived small interfering RNA-triggered DNA methylation. Plant Physiology 160, 990–999. 22846193 10.1104/pp.112.200279 PMC3461571

[CIT0085] Xia S , Xiao L, Gannon P, Li X. 2010. RFC3 regulates cell proliferation and pathogen resistance in Arabidopsis. Plant Signaling & Behavior 5, 168–170. 20023430 10.4161/psb.5.2.10526 PMC2884126

[CIT0086] Yang H , Lu P, Wang Y, Ma H. 2011. The transcriptome landscape of Arabidopsis male meiocytes from high-throughput sequencing: the complexity and evolution of the meiotic process. The Plant Journal 65, 503–516. 21208307 10.1111/j.1365-313X.2010.04439.x

[CIT0087] Ye J , Zhang Z, Long H, Zhang Z, Hong Y, Zhang X, You C, Liang W, Ma H, Lu P. 2015. Proteomic and phosphoproteomic analyses reveal extensive phosphorylation of regulatory proteins in developing rice anthers. The Plant Journal 84, 527–544. 26360816 10.1111/tpj.13019

[CIT0088] Ye J , Zhang Z, You C, Zhang X, Lu J, Ma H. 2016. Abundant protein phosphorylation potentially regulates Arabidopsis anther development. Journal of Experimental Botany 67, 4993–5008. 27531888 10.1093/jxb/erw293 PMC5014169

[CIT0089] Yelina NE , Lambing C, Hardcastle TJ, Zhao X, Santos B, Henderson IR. 2015. DNA methylation epigenetically silences crossover hot spots and controls chromosomal domains of meiotic recombination in Arabidopsis. Genes & Development 29, 2183–2202. 26494791 10.1101/gad.270876.115 PMC4617981

[CIT0090] Yi J , Kim SR, Lee DY, Moon S, Lee YS, Jung KH, Hwang I, An G. 2012. The rice gene DEFECTIVE TAPETUM AND MEIOCYTES 1 (DTM1) is required for early tapetum development and meiosis. The Plant Journal 70, 256–270. 22111585 10.1111/j.1365-313X.2011.04864.x

[CIT0091] Yuan TL , Huang WJ, He J, Zhang D, Tang WH. 2018. Stage-specific gene profiling of germinal cells helps delineate the mitosis/meiosis transition. Plant Physiology 176, 1610–1626. 29187566 10.1104/pp.17.01483 PMC5813559

[CIT0092] Zaidi MA , O’Leary SJB, Gagnon C, et al. 2020. A Triticale tapetal non-specific lipid transfer protein (nsLTP) is translocated to the pollen cell wall. Plant Cell Reports 39, 1185–1197. 32638075 10.1007/s00299-020-02556-6

[CIT0093] Zhai J , Zhang H, Arikit S, Huang K, Nan GL, Walbot V, Meyers BC. 2015. Spatiotemporally dynamic, cell-type-dependent premeiotic and meiotic phasiRNAs in maize anthers. Proceedings of the National Academy of Sciences, USA 112, 3146–3151. 10.1073/pnas.1418918112 PMC4364226 25713378

[CIT0094] Zhang W , Sun Y, Timofejeva L, Chen C, Grossniklaus U, Ma H. 2006. Regulation of Arabidopsis tapetum development and function by *DYSFUNCTIONAL TAPETUM1* (*DYT1*) encoding a putative bHLH transcription factor. Development 133, 3085–3095. 16831835 10.1242/dev.02463

[CIT0095] Zhang YC , Lei MQ, Zhou YF, et al. 2020. Reproductive phasiRNAs regulate reprogramming of gene expression and meiotic progression in rice. Nature Communications 11, 6031. 10.1038/s41467-020-19922-3 PMC7695705 33247135

[CIT0096] Zhang ZB , Zhu J, Gao JF, et al. 2007. Transcription factor AtMYB103 is required for anther development by regulating tapetum development, callose dissolution and exine formation in Arabidopsis. The Plant Journal 52, 528–538. 17727613 10.1111/j.1365-313X.2007.03254.x

[CIT0097] Zhao DZ , Wang GF, Speal B, Ma H. 2002. The excess microsporocytes1 gene encodes a putative leucine-rich repeat receptor protein kinase that controls somatic and reproductive cell fates in the Arabidopsis anther. Genes & Development 16, 2021–2031. 12154130 10.1101/gad.997902 PMC186413

[CIT0098] Zheng S , Li J, Ma L, Wang H, Zhou H, Ni E, Jiang D, Liu Z, Zhuang C. 2019. OsAGO2 controls ROS production and the initiation of tapetal PCD by epigenetically regulating OsHXK1 expression in rice anthers. Proceedings of the National Academy of Sciences, USA 116, 7549–7558. 10.1073/pnas.1817675116 PMC6462063 30902896

[CIT0099] Zhu J , Chen H, Li H, Gao JF, Jiang H, Wang C, Guan YF, Yang ZN. 2008. *Defective in Tapetal Development and Function 1* is essential for anther development and tapetal function for microspore maturation in Arabidopsis. The Plant Journal 55, 266–277. 18397379 10.1111/j.1365-313X.2008.03500.x

