# Supplementary material for: The proteome of developing barley anthers during meiotic prophase I
Source: J Exp Bot. 2021 Nov 10;73(5):1464–82. doi: 10.1093/jxb/erab494 (PMC8890616; doi:10.1093/jxb/erab494)
Supplement: erab494_suppl_Supplementary_Figures_S1-S3 [file erab494_suppl_supplementary_figures_s1-s3.pdf]

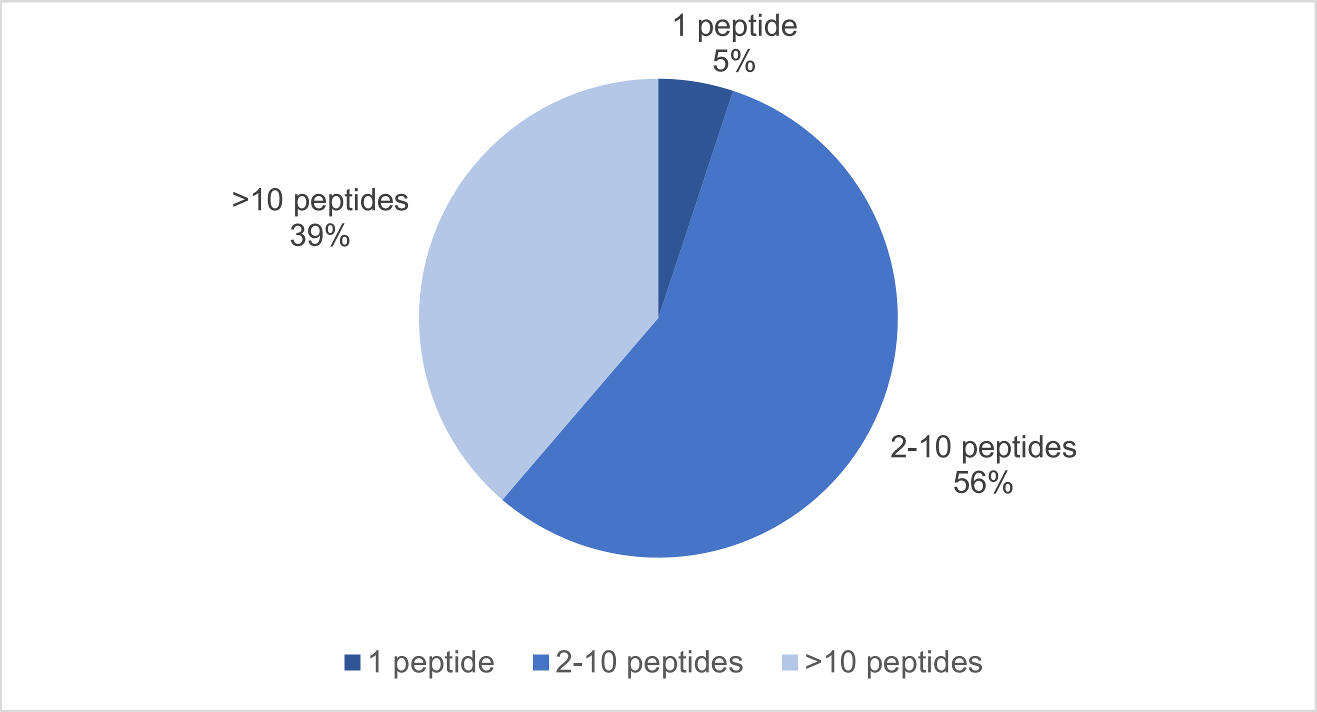

Supplementary Figure 1. Percentage of BaAP proteins identified with 1, more than 2 and more than 10 peptides

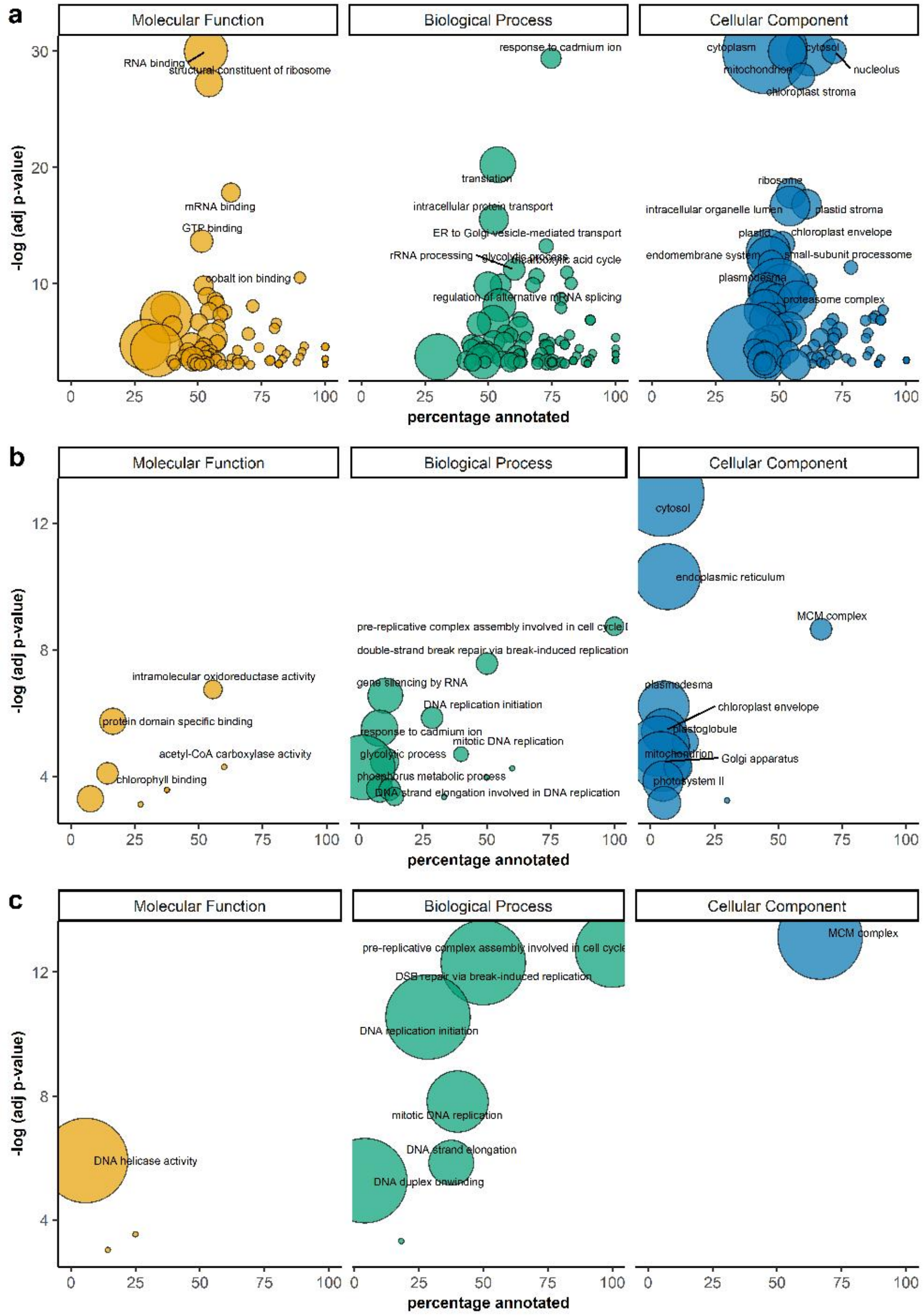

Supplementary Figure 2. Gene ontology enrichment analysis of all BaAP proteins (a), all differentially abundant proteins (DAPs) (b) and DAPs identified in comparison between stages 0.9 mm and 1.0 mm (c). Size of bubbles correspond to total number of proteins associated with the GO term.

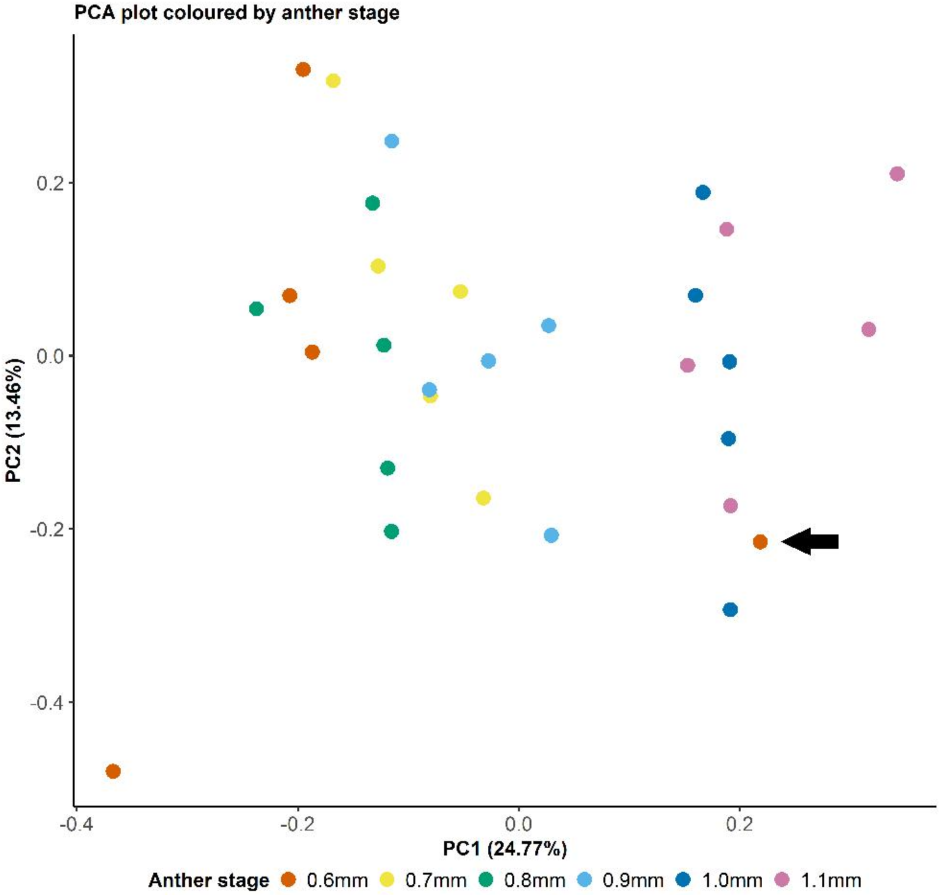

Supplementary Figure 3. Sample clustering by Principal Component Analysis (PCA) by anther stage. Arrow represents an outlier sample from 0.6mm anther replicate 5
